# *Drosophila* MARF1 ensures proper oocyte maturation by regulating nanos expression

**DOI:** 10.1371/journal.pone.0231114

**Published:** 2020-04-03

**Authors:** Shinichi Kawaguchi, Mizuki Ueki, Toshie Kai

**Affiliations:** Graduate School of Frontier Biosciences, Osaka University, Suita, Osaka, Japan; University of Cambridge, UNITED KINGDOM

## Abstract

Meiosis and oocyte maturation are tightly regulated processes. The meiosis arrest female 1 (*MARF1*) gene is essential for meiotic progression in animals; however, its detailed function remains unclear. In this study, we examined the molecular mechanism of *dMarf1*, a *Drosophila* homolog of *MARF1* encoding an OST and RNA Recognition Motif (RRM) -containing protein for meiotic progression and oocyte maturation. Although oogenesis progressed in females carrying a *dMarf1* loss-of-function allele, the *dMarf1* mutant oocytes were found to contain arrested meiotic spindles or disrupted microtubule structures, indicating that the transition from meiosis I to II was compromised in these oocytes. The expression of the full-length *dMarf1* transgene, but none of the variants lacking the OST and RRM motifs or the 47 conserved C-terminal residues among insect groups, rescued the meiotic defect in *dMarf1* mutant oocytes. Our results indicate that these conserved residues are important for dMarf1 function. Immunoprecipitation of Myc-dMarf1 revealed that several mRNAs are bound to dMarf1. Of those, the protein expression of *nanos* (*nos*), but not its mRNA, was affected in the absence of *dMarf1*. In the control, the expression of Nos protein became downregulated during the late stages of oogenesis, while it remained high in *dMarf1* mutant oocytes. We propose that *dMarf1* translationally represses *nos* by binding to its mRNA. Furthermore, the downregulation of Nos induces *cycB* expression, which in turn activates the CycB/Cdk1 complex at the onset of oocyte maturation.

## Introduction

Oogenesis is the process of forming a mature female gamete, called an oocyte. This process is tightly regulated to ensure proper fertilization and embryogenesis. *Drosophila melanogaster* is an ideal model organism for the detailed study of oogenesis. *Drosophila* females have one pair of ovaries that consist of 18–20 ovarioles (reviewed in [1–3]). In germarium—a structure at the anterior tip of the ovariole—two or three germline stem cells (GSCs) are maintained in the undifferentiated status in a niche environment. Following the asymmetric division of a GSC into two daughter cells, one cell remains as a GSC in the niche, whereas the other daughter cell (cystoblast) departs the niche to commence differentiation. A cystoblast divides four times to produce a 16-cell germline cyst. Among the 16 cells, one develops into an oocyte and the remaining 15 cells become nurse cells. The nurse cells form polyploid nuclei for active gene expression to deliver RNAs and proteins into the oocyte through a cytoplasmic bridge known as the ring canal. During stage 10 of oogenesis, the nurse cells begin to shrink and dump their contents into the transcriptionally dormant oocyte, which is arrested at meiotic prophase I. Stage 13 of oogenesis is characterized by the commencement of oocyte maturation, release of the first meiotic arrest (prophase I), degradation of nuclear membrane, chromosome condensation, and formation of metaphase spindles. At stage 14, the oocyte becomes arrested again at metaphase I and is competent for fertilization.

*Drosophila melanogaster* Polo kinase plays an important role in inducing oocyte maturation. Its activity is tightly regulated by several proteins, including Endos, Matrimony, and Greatwall (reviewed in [2]). The activity of Polo kinase increases at the onset of oocyte maturation. Polo kinase phosphorylates and activates Twine phosphatase (Cdc25); in turn, Twine dephosphorylates and activates cyclin-dependent kinase 1 (Cdk1), a subunit of M-phase promoting factor (MPF). MPF is a complex composed of Cdk1 and one of the three known cyclins—Cyclin A, B, or B3—and is formed during meiosis [4]. The CycA/Cdk1 complex plays an important role in nuclear envelope breakdown (NEB) and release of prophase I arrest, the first meiotic arrest point [5]. The release of prophase I arrest upregulates CycB protein expression and induces the formation of CycB/Cdk1 complex to promote oocyte maturation and meiotic spindle formation. After progressing to metaphase I, the oocytes undergo a second arrest. Finally, ovulation releases this second meiotic arrest, and the oocytes are further activated for fertilization [2]. Although each process has been well-studied, the detailed molecular mechanism triggering the maturation of oocytes remains unclear.

The *MARF1* (Meiosis arrest female 1) gene was first reported to be essential for meiotic progression in mouse [6]. The *MARF1* mutant female mouse is sterile due to its disability to release the first meiotic arrest following gonadotropin-induced oocyte maturation. The catalytic subunit of protein phosphatase 2A (PP2A) is upregulated in the absence of MARF1 activity, and results in the reduction of MPF activity and failure of oocyte maturation. Although the molecular mechanism associating ectopic activity of PP2A and defective meiotic progression remains elusive, mouse MARF1 functions to maintain the protein phosphorylation cascade for meiotic maturation. In addition, Yao et al. have recently reported that the ribonuclease activity of the NYN nuclease domain of mouse MARF1 regulates meiotic progression and genome integrity in oocytes [7]. However, the NYN nuclease domain is not conserved in the *Drosophila* homolog of MARF1. In order to understand the molecular mechanism of meiotic progression and oocyte maturation, we investigated the activities of dMarf1, a *Drosophila* homolog of MARF1, in this study. Although the mutant females carrying a dMarf1 loss-of-function allele laid eggs, they never hatched due to incomplete meiosis.

During our study, Fukunaga’s group at Johns Hopkins reported a characterization of *dMarf1* [8]; dMarf1 was required for the meiosis completion and interacted with CCR4-NOT deadenylase. They also found that *cyclin A* was one of the targets of the dMarf1/CCR4-NOT function and proposed the mechanism of the temporal regulation of cyclin A reduction during late oogenesis. In this paper, we propose an additional layer of dMarf1 function. We identified a few mRNAs including *nanos* (*nos*) bound by dMarf1. Nos protein, which functions as a translational repressor of CycB, is downregulated during late oogenesis in the control. By contrast, high expression of Nos was found even in the mature oocytes of the *dMarf1* mutant females. Our results suggest that *dMarf1* can repress *nos* to induce *cycB* expression, and activate CycB-Cdk1 complex (MPF) at the onset of oocyte maturation.

## Materials and methods

### Fly strains

All fly stocks were maintained at 25°C or 18°C. Either the *yw* strain or the respective heterozygote was used as a control. A TRiP line, HMS04468 (BL#57024, Bloomington Drosophila Stock Center, IN, USA), was used to knockdown *dMarf1* expression. The following drivers were used for transgene or RNAi expression: *NGT40* and *nosGal4-VP16*, *Matα Gal4-VP16* (BL#7063) (for germline cells) and *traffic jam-Gal4* (for somatic follicle cells). A loss-of-function allele of *dMarf1*, *dMarf1*^*KO321*^, was generated using the CRISPR-Cas9 system (details are described in the S1 Protocol). The resultant mutant strain was back-crossed three times with the wild-type fly, and maintained over CyO balancer chromosome. To generate flies expressing *dMarf1* transgene variants, the coding sequences of full-length dMarf1 or its truncated mutants (ΔN, ΔOST, ΔRRM, ΔC, and ΔC47) were PCR amplified using the cDNA clone RE61489 as a template. Triple repeats of the Myc-epitope tag were added to the N-terminal region. Purified PCR products were assembled using the In-Fusion system (Takara Bio, Japan) with an *XbaI*-digested pUASp-attB vector (Drosophila Genomics Resource Center). The resultant plasmids were injected into embryos for insertion at the phiC31 integration site—attP-3B—at 65B2 (BL#32543) using the standard protocol [9,10]. The primers used in this study are shown in S5 Table.

### Isolation of egg chambers

Female flies were fed with wet-yeast paste for 2 to 3 days, and their ovaries were dissected in oocyte isolation buffer (100 mM HEPES-Na, pH 7.4, 55 mM NaOAc, 40 mM KOAc, 110 mM sucrose, 1.2 mM MgCl_2_, 1 mM CaCl_2_) to prevent oocyte activation [11] Egg chambers were dissected from ovarioles by using tungsten needles under the dissection microscope. Stages of egg chambers were assigned based on the standard King’s criteria [12]. Each group of egg chambers were collected into a 1.5 ml tubes, homogenized by the plastic pestle, and examined for protein expression by Western blotting analysis.

### Immunostaining

Germaria and egg chambers (stage 1–12) were immunostained as described previously [13]. For staining stage 13–14 egg chambers, 10–20 ovaries were dissected from the adult females and immediately transferred to 1.5 ml tube containing ~1 ml of 100% methanol. To disrupt the eggshell and facilitate antibody penetration, ovaries were sonicated 20 times for 0.5 second each by using the sonicator (TOMY UD-201) with the following settings: Output 2 and Duty 50. The sonicated ovaries were washed with 100% methanol and sequentially incubated at 25°C in 90% methanol for 60 min, in 75% methanol/PBS for 30 min, in 50% methanol/PBS for 30 min, in PBS for 30 min, and in PBS containing 1.0% Triton X-100 for 60 min, followed by the conventional immunostaining as described previously [13]. For immunostaining of unfertilized eggs, females were mated with *can*^*12*^ homozygous mutant males [14], which cannot produce mature sperms. Thus, females were induced to lay unfertilized eggs. Immunostaining was performed using the following primary antibodies: mouse monoclonal anti-Myc (1:400 dilution) (9E10, Wako, Japan), mouse anti-α-tubulin (1:400) (DM1A, Santa Cruz Biotechnology, TX, USA), and rabbit anti-phospho-histone H3 (1:400) (#06–570, Merck Millipore, MA, USA), and the following secondary antibodies: Alexa Fluor 488-, or 555-conjugated goat anti-rabbit, and anti-mouse IgG (1:400) (Thermo Fisher Scientific, MA, USA). The chromosomes and actin filament were stained with 4′,6-Diamidine-2′-phenylindole (DAPI, Sigma-Aldrich, MO, USA) and rhodamine-phalloidin (Cytoskeleton, CO, USA), respectively. Images were acquired using an Olympus FV1000 or Zeiss LSM780 confocal microscope.

### Immunoprecipitation

Myc-tagged full-length dMarf1 (Myc-dMarf1-FL) was expressed in germline cells by crossing with the *Matα* driver. Approximately 300 ovaries were collected and homogenized using a hand pestle in the oocyte isolation buffer containing proteinase inhibitor cocktail (Roche, Switzerland) and 0.1% Triton X-100 (Wako). The lysate was cleared by two rounds of centrifugation at 20,000 Relative Centrifugal Force (RCF). Myc-dMarf1-FL protein was immunoprecipitated from the cleared ovary lysate using anti-c-Myc antibody-conjugated magnetic beads (Thermo Fisher Scientific). Anti-HA antibody magnetic beads (Thermo Fisher Scientific) were used as a negative control. After washing the beads with phosphate-buffered saline (PBS) containing 0.1% Triton X-100, TRIzol^TM^ (Invitrogen, OR, USA) was added to extract the bound RNA.

### Generation of anti-dMarf1 antibody

The C-terminal nucleotide sequence of dMarf1 (901–1305 residues) was amplified and ligated into pENTR^TM^/D-TOPO (Invitrogen), and recombined into pDEST17 to generate a His-tag fusion protein, His-dMarf1-C, using the Gateway system (Invitrogen). The His-dMarf1-C protein expressed in *E*. *coli* was purified using Ni Sepharose^TM^ 6 beads (GE Healthcare) according to the manufacturer’s protocol. The purified protein combined with complete adjuvant was injected into guinea pigs. Serum was obtained by centrifugation and heat inactivated at 56°C for 30 min.

### Quantitative RT-PCR

RNA was extracted from the ovaries or immunoprecipitates using TRIzol^TM^ reagent (Invitrogen), according to the manufacturer's protocol. The purified RNA (~1 μg) was treated with DNase I (New England Biolabs, MA, USA) to remove any contaminating genomic DNA. DNase I was inactivated at 70°C for 10 min in the presence of EDTA, and the complementary DNA was synthesized using superscript III (Invitrogen). Real-time PCR was performed using Fast SYBR Green system (Thermo Fisher Scientific) with StepOnePlus (Applied Biosystems, CA, USA). *Act5C* was used for normalization.

### mRNA sequencing and analyses

Total RNAs were extracted from hand-dissected ovaries from *dMarf1*^*KO321*^/*CyO* heterozygous and *dMarf1*^*KO321*^ homozygous females using TRizol (ThermoFisher) in accordance with the manufacturer’s instruction. Quality of the purified RNA was examined by Agilent 2100 Bioanalyzer (Agilent) prior to the library construction. RNAs with poly(A) tails were enriched by using oligo-dT beads. RNAs containing poly(A) tails were purified with oligo-dT beads, fragmented and reverse transcribed. Adapters were ligated and the libraries were amplified by PCR. DNA fragments with small size (less than 200 bp) were removed by AMPure XP beads (Beckman Coulter). Libraries were sequenced with Illumina HiSeq sequencer (Illumina) on a 100 cycle Single End Read sequencing run. The cDNA library preparation and next-generation sequencing were performed at Hokkaido System Science (Hokkaido, Japan). Total reads obtained by the Illumina HiSeq sequencing were 23,867,733 and 23,608,608 reads for *dMarf1*^*KO321*^ heterozygous control and homozygous mutant, respectively. Reads were mapped to the *Drosophila* genome (Release 6) using the TopHat2 program [15]. The mapping rate was 88.8% and 88.6% for *dMarf1*^*KO321*^ heterozygous control and homozygous mutant, respectively. The differentially-expressed genes were detected by Cuffdiff program from the Cufflinks package [16].

### RNA-IP-seq and analyses

Myc-dMarf1 was immunoprecipitated from the ovarian lysate using anti-c-Myc antibody-conjugated magnetic beads as described in Materials and Methods. Immunoprecipitate with anti-HA antibody-conjugated magnetic beads was used as a negative control. RNAs bound to the magnetic beads were isolated by TRizol (ThermoFisher) in accordance with the manufacturer’s instruction. Quality of the purified RNA was checked by Agilent 2100 Bioanalyzer prior to the library construction. The following RNA purification and the library construction were conducted as described above. Libraries were sequenced with Illumina HiSeq sequencer on a 150 cycle Pair-End Read sequencing. The cDNA library preparation and next-generation sequencing were performed at Gene-Nex (Japan). Total reads obtained by the Illumina HiSeq sequencing were 14,273,308 pairs and 10,686,509 pairs for Myc-dMarf1 bound and control fraction, respectively. Among reads, low-quality reads and adaptor sequences (PE1; TACACTCTTTCCCTACACGACGCTCTTCCGATCT, PE1_rc; AGATCGGAAGAGCGTCGTGTAGGGAAAGAGTGTA, PE2; GTGACTGGAGTTCAGACGTGTGCTCTTCCGATCT, PE2_rc; AGATCGGAAGAGCACACGTCTGAACTCCAGTCAC) were trimmed by Trimmomatic software [17]. Around 2.6% reads were trimmed in both libraries. The resulting reads were mapped to the *Drosophila* genome (Release 6) using the TopHat2 program [15]. The mapping rate was 89.3% and 89.8% for Myc-dMarf1 bound and control fraction, respectively. The differentially expressed genes were detected by Cuffdiff program from the Cufflinks package [16].

### Quantitative proteome analysis

The heterozygous and homozygous ovaries of *dMarf1*^*KO321*^ were dissected in oocyte isolation buffer. 50 of stage 14 egg chambers were picked up into 1.5 ml tubes, and the buffer was replaced with 50 μl of 50 mM Tris-Cl (pH 8.0) buffer containing 2% deoxycholate and proteinase inhibitor cocktail. The egg chambers were homogenized by the plastic pestle, and the lysates were cleared by centrifugation at 20,000 RCF for 1 min at 4°C. Homogenization and centrifugation were repeated twice more. After third homogenization, lysates were further cleared by centrifugation at 20,000 RCF for 15 min at 4°C. The supernatants (~30 μl) were collected in low-protein binding tubes (Eppendorf, Germany). The protein concentrations of the lysates were between 0.95 to 1.37 μg/ml. Proteins extracted from egg chambers were reduced, alkylated, and digested with trypsin. The peptides were labeled using the Tandem Mass Tag^TM^ system (Thermo Fisher Scientific). The pooled peptides were separated using the UltiMate 3000 RSLCnano system and analyzed by Q-Exactive mass spectrometry (Thermo Fisher Scientific). The differential protein expression levels between control and mutant were determined based on the signal strengths of the corresponding peptides detected for each protein. We had two biological replicates for each control and mutant samples. P-value was calculated for proteins with more than two peptides detected by using a standard software, Scaffold (Proteome Software, Oregon) and shown in S3 Table.

### *In vitro* oocyte activation

Mature but inactive egg chambers were isolated from dissected ovaries in the oocyte isolation buffer at 25°C. Oocyte activation was initiated by exchanging the isolation buffer with a hypotonic buffer (20 mM PIPES, 10 mM NaCl, 50 mM KCl, 5% PEG6000, 2 mM CaCl_2_, pH 6.4) [11,18]. After incubation for 30 min, the egg chambers were treated with 50% bleach for 2 min to lyse the inactive egg chambers. The resistant egg chambers were considered activated oocytes.

### Western blotting

Protein lysates from ovaries (one-half ovary equivalent per lane) were electrophoresed on a 7.5% or 10% sodium dodecyl sulfate-polyacrylamide gel. The following primary antibodies were used: guinea pig anti-dMarf1 (1:1,000, this study), mouse anti-Myc 9E10 (1:5000, Wako), mouse anti-CycA (1:1000, DSHB, IN, USA), mouse anti-CycB (1:1000, DSHB), rabbit anti-Nanos (1:1000, from Dr. Nakamura), rabbit anti-Dhd (1:1000, from Dr. Loppin), mouse anti-Polo (1:400, from Dr. Glover), mouse anti-glorund (1:1000, DSHB), and mouse anti-αTubulin DM1A (1:1000, Santa Cruz Biotechnology). Immunoreactive bands were visualized using HRP-conjugated goat anti-guinea pig (1:1000, Dako, Denmark), anti-rabbit, or anti-mouse secondary antibodies (1:3000, Bio-Rad, CA, USA), and immunoblots were detected using LAS-1000 (GE Healthcare) with an ECL chemiluminescent substrate (Thermo Fisher Scientific).

### Data deposition

The next generation sequencing data shown in this study are deposited to the DDBJ sequence Read Archive (DRA). The submission IDs for RNA-seq and RIP-seq data are SSUB012974 and SSUB012977, respectively.

## Results

### MARF1 is an RRM and OST motif-containing protein conserved in animals

**Fig 1:**
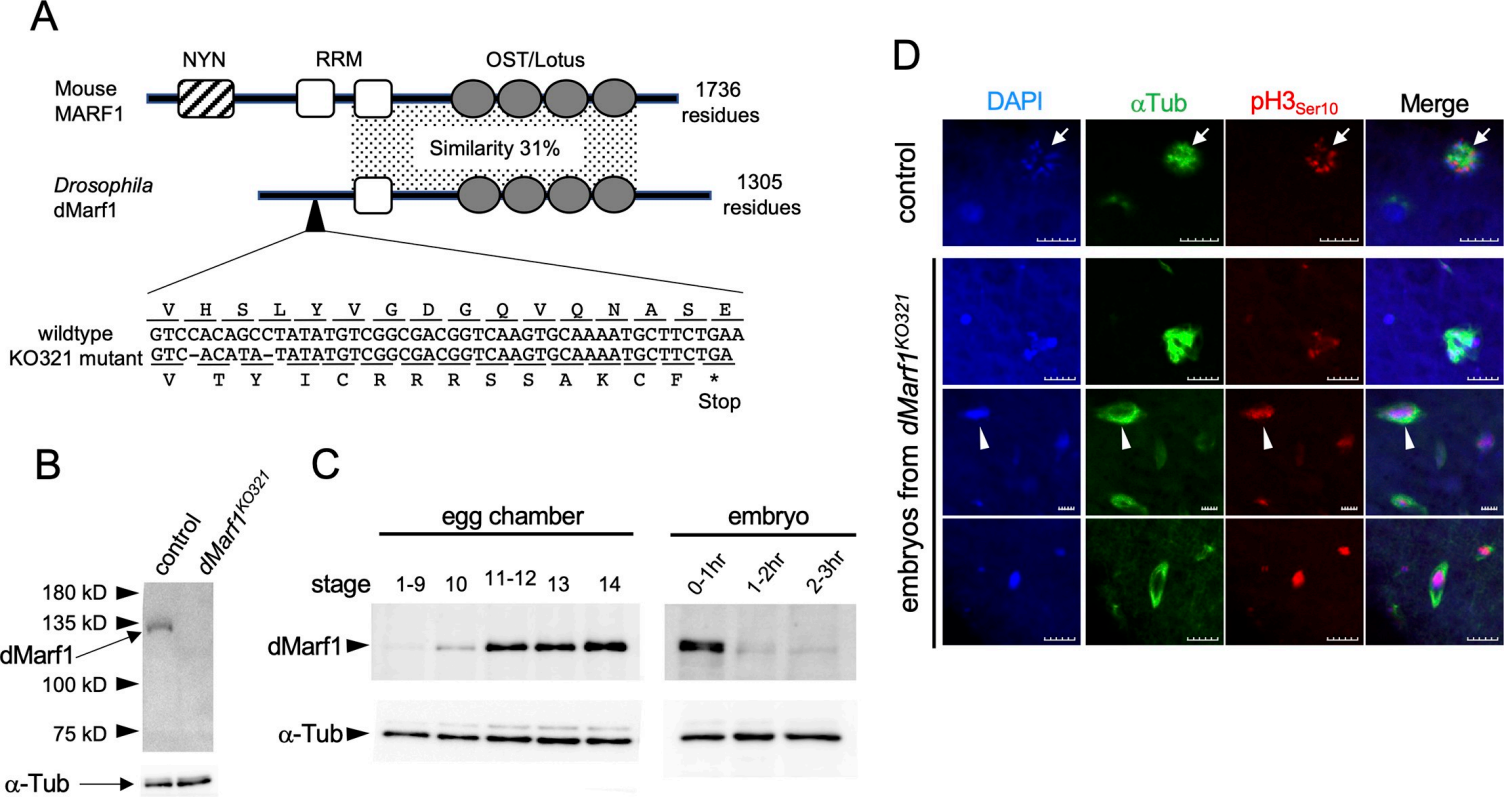
dMarf1 is a conserved protein and functions in the late stages of oogenesis. (A) Schematic model of mouse MARF1 and *Drosophila* dMarf1 proteins. NYN, RRM, and OST/Lotus correspond to Nedd4BP1 and YacP nuclease domain, RNA Recognition Motif, and Oskar-TDRD5/7 domain, respectively. The polypeptide sequence similarity in the conserved domain region is shown. The loss-of-function allele, *KO321*, contains a two-nucleotide deletion, which results in premature termination of translation. (B) Western blotting of ovarian lysates from the control and *dMarf1*^*KO321*^ homozygous mutant females. Each lane contains equivalents of 0.5 ovaries. (C) Western blotting showing temporal regulation of dMarf1 protein expression at different stages of egg chambers and embryo development. α-Tubulin was used as a loading control. dMarf1 expression was upregulated in stage 12 egg chambers, and immediately reduced after the onset of embryogenesis. (D) Embryos were collected from the heterozygous control and *dMarf1*^*KO321*^ homozygous mutant mothers were collected for 6-hrs after egg laying and stained with DAPI (blue), anti-α-Tubulin (green), and anti-phospho-Histone H3 (red). Young control embryos (0-2-h old) exhibit the characteristic rosette structure containing three pronuclei, while 0–6 h-old embryos from the mutant mothers do not. Scale bar, 10 μm.

The genome of *Drosophila melanogaster* was screened for the mouse homolog of MARF1 using the method of polypeptide similarity search. The protein encoded by the *CG17018* gene showed the highest score. The full-length CG17018 polypeptide consists of 1,305 amino acid residues and shares 31% sequence identity with mouse MARF1 in their middle regions that contain the RNA Recognition Motif (RRM) and OST/Lotus domains (Fig 1A). The OST/Lotus domain is highly conserved from bacteria to humans, and proteins harboring this domain are exclusively expressed in germline cells of animals [19,20]. The presence of multiple OST/Lotus domains is a characteristic of the MARF1 family. MARF1 homologs in most animals, except *Drosophila*, contain a nuclease domain (NYN) (Fig 1A and S1 Fig). Hereafter, CG17018 is referred to as dMarf1, a *Drosophila* homolog of MARF1.

Secondary structure prediction was performed to examine the structural features of dMarf1. While the RRM and OST domains are highly structured, the N-terminal region of approximately 200 residues and C-terminal region of approximately 300 residues were predicted to be unstructured (S2 Fig). The unstructured C-terminal region is not evolutionary conserved among all animal groups. However, it is well conserved among higher animal groups such as humans, mouse, *Xenopus*, and Zebrafish (S3 Fig). Moreover, the C-terminal region of human MARF1 is proposed to be a binding site for Ge-1, a key component of the decapping complex [21]. The C-terminal sequence of dMarf1 is different from those of higher animals. However, in contrast to the highly diverse N-terminal region, the C-terminal region consisting of 50 amino acids is well conserved among insects (S4 Fig). This finding suggests that the C-terminal regions is functionally important for dMarf1.

### dMarf1 functions during late oogenesis

We generated a loss-of-function allele *dMarf1*^*KO321*^ using the CRISPR-Cas9 system to study the *in vivo* function of dMarf1. Genome editing resulted in a two-nucleotide deletion in the N-terminal region of dMarf1 (Fig 1A), causing a frameshift mutation and premature termination of translation. *dMarf1*^*KO321*^ males did not show any defect; normal egg hatching was observed when the control wild-type females were mated with *dMarf1*^*KO321*^ males (S5A Fig). By contrast, *dMarf1*^*KO321*^ females were sterile. Although the mutant females produced a normal number of eggs upon mating with control males, none of them hatched (S5B Fig). We further confirmed this defect by downregulating *dMarf1* expression using small RNA-based strategy [22]. The TRiP line flies carrying the *HMS04468* transgene to express shRNA complementary to *dMarf1* were crossed with two different germline driver lines—*NGT40* and *nosGal4-VP16—*to drive expression from the germarium to the late stages of egg chambers, and *Mat*α*-Gal4* in germline cells after stage 2 of egg chambers [23,24]. We also used a somatic driver, *traffic jam-Gal4* (*tj-Gal4*), as a control. The mutant females in which *dMarf1* expression was downregulated using either germline driver line laid a similar number of eggs compared to the control females (S5C Fig). However, the eggs from the mutant females did not hatch (S5D Fig). By contrast, the downregulation of *dMarf1* with somatic *tj-Gal4* did not cause any defects. These results suggest that *dMarf1* plays an important role in germline cells after stage 2 of oogenesis.

Next, we generated an antibody against dMarf1 to examine the protein expression of dMarf1. Western blotting revealed the presence of a unique band in the control ovary lysate that was absent in the *dMarf1*^*KO321*^ homozygous mutant ovary lysate (Fig 1B). This result indicated that the antibody specifically targets dMarf1 in the control ovaries and dMarf1 expression is lost in the mutant ovaries. Consistent with its predicted size, the apparent molecular weight of dMarf1 was approximately 130 kDa. dMarf1 was first detected in stage 10 of oogenesis, and its expression increased until stage 14 (Fig 1C). dMarf1 expression was detected during the initial 2 h after egg laying, and immediately decreased thereafter. These results suggest that *dMarf1* functions in the late stages of oogenesis.

Because the eggs laid by *dMarf1*^*KO321*^ females did not hatch (S5B Fig), we next examined meiotic progression in the absence of *dMarf1*^*KO321*^ by immunostaining α-tubulin and phospho-histone H3 (pH3), an M-phase marker. The fertilized eggs of the control females exhibited the characteristic rosette structure containing three pronuclei, indicating the completion of meiosis (arrow, Fig 1D). In contrast, the eggs of the *dMarf1*^*KO321*^ females contained fragmented or over-replicated chromosomes that were often surrounded with microtubules, but not the rosette structure (arrowhead, Fig 1D). These results suggest that meiosis was incomplete in the *dMarf1*^*KO321*^ oocytes.

### *dMarf1* is required for meiosis I to II transition

**Fig 2:**
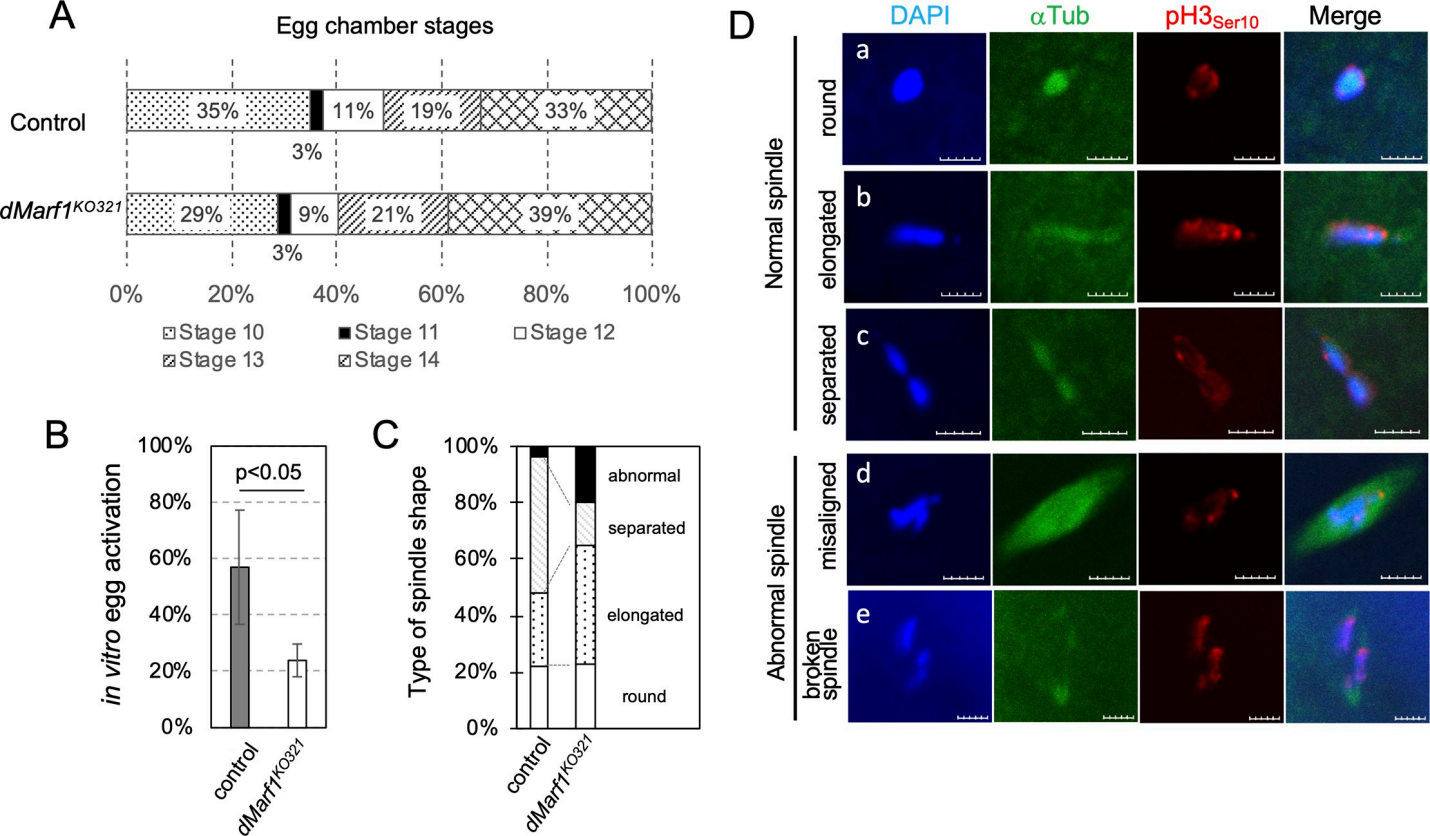
*dMarf1* functions during the late stages of oogenesis. (A) The ratio of each stage is plotted for the egg chambers from heterozygous control ovaries (upper bar) and *dMarf1*^*KO321*^ ovaries (lower bar), respectively. Stages of egg chambers were assigned based on the standard King’s criteria [12]. (B) *In vitro* activation of stage 14 egg chambers. The average ratio ± standard deviation is shown for the control (n = 94) and *dMarf1*^*KO321*^(n = 158) ovaries (p < 0.05; Student’s t-test). (C-D) Meiotic spindles in stage 14 egg chambers. Most spindles in the control were round, long, or separated. By contrast, 20% of stage 14 egg chambers in the *dMarf1*^*KO321*^ ovaries contained abnormal (broken or mis-aligned) spindles. The ratio of each spindle shape type is plotted as a bar graph in (C) (n = 58 for control, and n = 91 for *dMarf1*^*KO321*^), and immunostaining of the corresponding spindle types are shown in (D). DAPI in blue, α-tubulin in green, phospho-histone H3 (an M-phase marker) in red. Scale bar, 5 μm.

Next, we examined late-stage oogenesis defects in *dMarf1*^*KO321*^ mutant ovaries. The egg chambers of heterozygous control and *dMarf1*^*KO321*^ ovaries were categorized based on the King’s criteria [12] and counted at each stage. The distribution of the egg chamber stages was not significantly different between the control and *dMarf1*^*KO321*^ ovaries (Fig 2A). In addition, *dMarf1*^*KO321*^ mutant females laid similar number of eggs compared to the control (S5B Fig). These results suggested that oogenesis progresses normally in the absence of *dMarf1* until stage 14. This result also suggests that *dMarf1* is not required for the release of prophase I arrest of stage 13 egg chamber, unlike mouse MARF1 that is required for the release of prophase I arrest of meiotic division (GV stage)[6].

To further examine whether *dMarf1* plays a role in the maturation process after the release of the first arrest point, we performed the egg activation assay *in vitro* [11]. Dehydrated and inactive stage 14 egg chambers were dissected from ovaries in a hypertonic buffer to retain dehydration, and subsequently activated by hydration in a hypotonic buffer [11]. Of all the control eggs, 60% were activated and showed resistance to bleach by crosslinking of vitelline membrane proteins in meiosis II (Fig 2B). By contrast, only 25% of the *dMarf1*^*KO321*^ mutant eggs were resistant to bleach, indicating the failure of vitelline membrane maturation in the absence of *dMarf1*. This result prompted us to examine meiotic spindles of the metaphase I-arrested oocytes. Symmetric spindles (round, long, and separated) were detected in 97% of the control oocytes at stage 14, and only 3% showed abnormally shaped spindles. However, 22% of the stage 14 *dMarf1*^*KO321*^ oocytes exhibited broken spindles or misaligned chromosomes (Fig 2C and 2D). These results suggest that *dMarf1* is required to stabilize meiosis I spindle structure.

**Fig 3:**
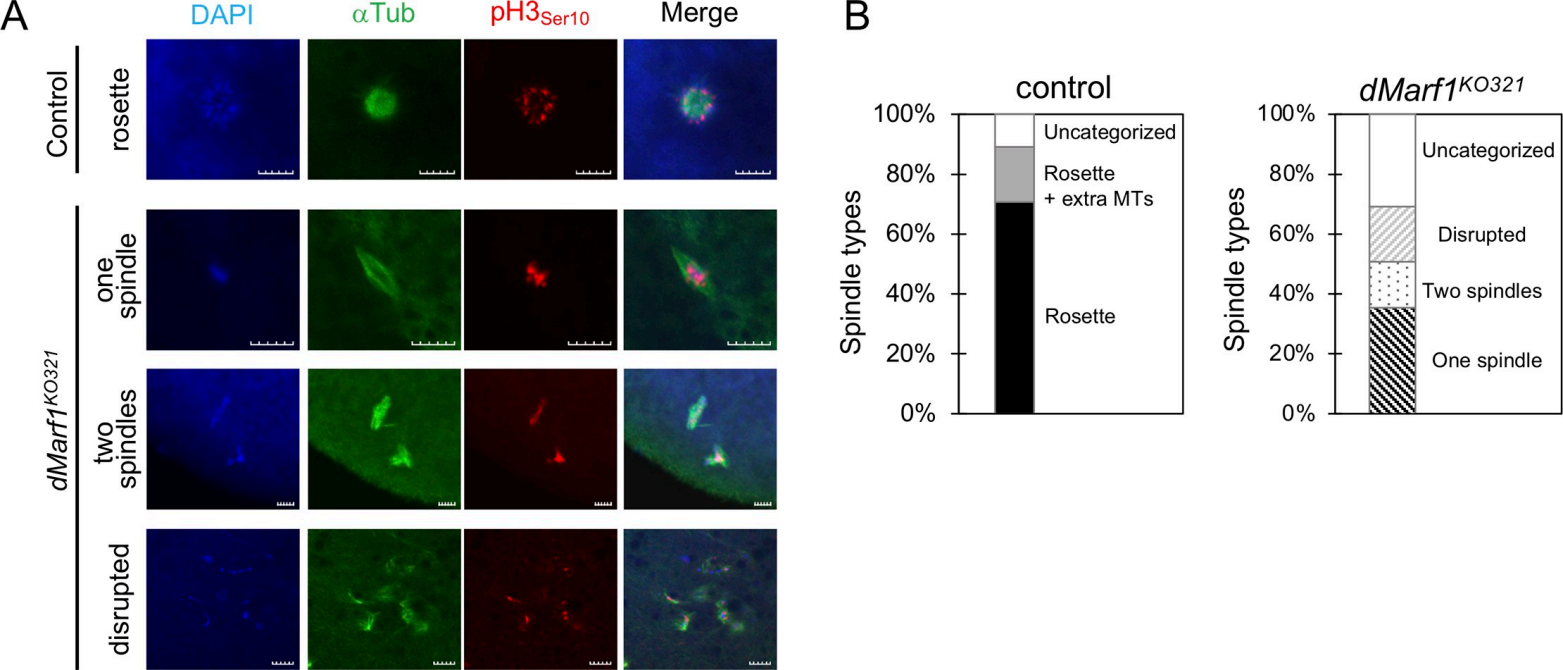
Meiosis I to II transition is compromised in *dMarf1*^*KO321*^ mutant females. (A-B) Meiotic products in the unfertilized egg from the heterozygous control or homozygous *dMarf1*^*KO321*^ females. The microtubule structures were classified into the following categories: control: rosette structure (a fused structure of pronuclei), rosette with two to four extra spindles, and an uncategorized structure; *dMarf1*^*KO321*^: one spindle, two spindles, disrupted spindles/microtubules, and an uncategorized structure. The ratio of each spindle type was plotted in a bar graph (B) (n = 181 for control, and n = 140 for *dMarf1*^*KO321*^), and immunostaining of the corresponding spindle type is shown in (A). DAPI in blue, α-tubulin in green, phospho-histone H3 (an M-phase marker) in red. Scale bar, 10 μm.

Furthermore, the *dMarf1* mutant eggs exhibited severe defects in meiosis I to II progression (Fig 3). Oocytes in a second arrest at metaphase I can be released from the arrest by ovulation to resume meiosis I to II progression without requiring fertilization. Of all the heterozygous control eggs, 71% showed the characteristic rosette structure with pronuclei and microtubules, indicating the completion of meiosis (Fig 3A, top panel, and B). In another 18%, two to four additional microtubules with the rosette structure were observed. However, no rosette structure was observed in the *dMarf1*^*KO321*^ mutant eggs (Fig 3A: lower panels). Among those, 36% of the mutant eggs showed a single metaphase I-like spindle, indicative of prolonged arrest state (Fig 3B), 15% showed two spindle-like structures that were not connected to each other as transiently observed during meiosis II [11], and 19% exhibited abnormal and disrupted spindle structures. In the *dMarf1*^*KO321*^ oocytes, fragmented or over-duplicated chromosomes were often surrounded by microtubules (Fig 3A: bottom panels). These results indicate that meiosis is incomplete and meiosis I to II transition is compromised in the absence of *dMarf1*.

### dMarf1 is expressed in nurse cells and deposited into ooplasm

**Fig 4:**
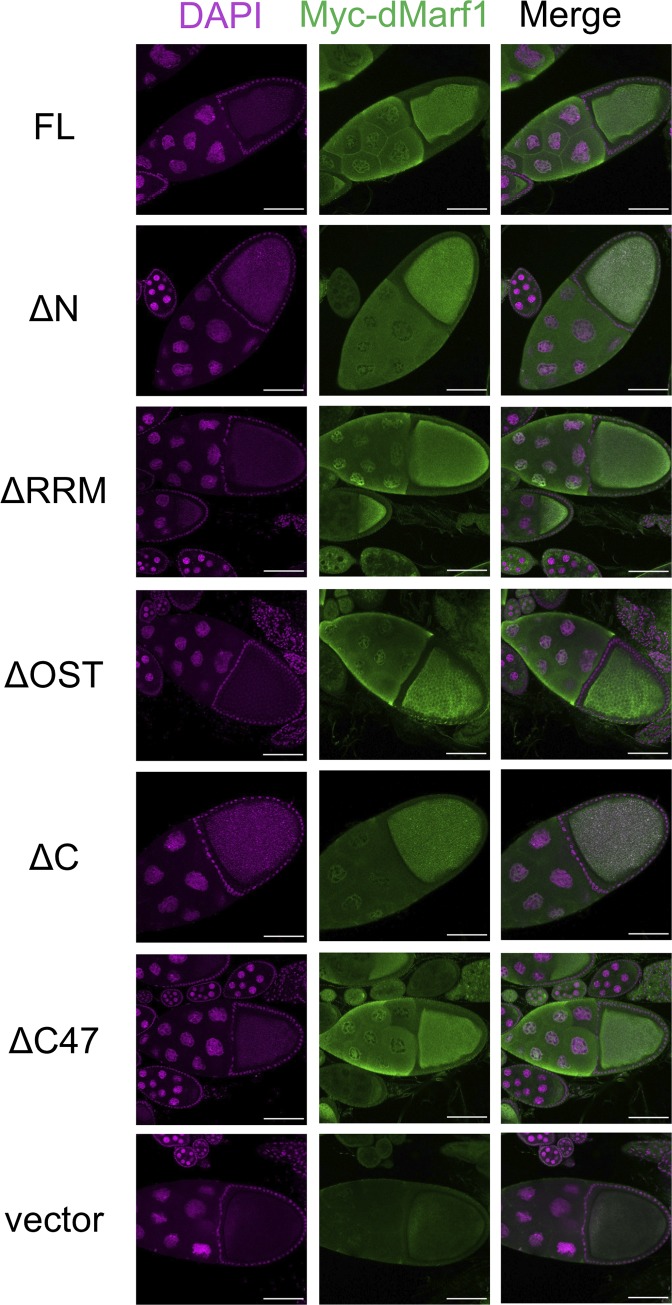
Localization of full-length or truncated variants of Myc-dMarf1 in germline cells. Full-length (FL) Myc-dMarf1 and its truncated variants, lacking 252 N-terminal amino acid residues (ΔN), RRM domain (ΔRRM), OST domain (ΔOST), C-terminal region (ΔC), and 47 C-terminal amino acid residues (ΔC47), were expressed in female germline cells and immunostained with an anti-Myc antibody. DAPI in magenta, α-Myc in green. Scale bar, 50 μm.

Western blotting revealed that dMarf1 expression increased in the late stages of oogenesis (Fig 1C). Hence, we analyzed the subcellular localization of dMarf1 in the egg chambers. Because the anti-dMarf1 antibody did not work for immunostaining, we used a transgenic fly expressing Myc-tagged full-length dMarf1 (Myc-dMarf1-FL). Myc-dMarf1-FL driven by a *Mat*α*-Gal4* germline driver was detected both in oocytes and nurse cells using the anti-Myc antibody (Fig 4). In nurse cells, the nuclear expression of Myc was slightly higher than its cytoplasmic expression, suggesting that dMarf1 is localized into the nucleus. The expression of Myc-dMarf1-FL in a *dMarf1*^*KO321*^ mutant background significantly increased the hatching rate of eggs (up to 17%); however, none of the *dMarf1*^*KO321*^ mutant eggs hatched (S6A Fig). Consistently, embryogenesis of the *dMarf1*^*KO321*^ mutant eggs was recovered, normal mitotic spindles were observed in the rescued eggs (S6B Fig), and 20% of the mutant eggs passed through the blastoderm within 6 h after egg laying. However, the *dMarf1*^*KO321*^ mutant eggs lacking Myc-dMarf1-FL expression did not show any sign of development (S6C Fig).

Next, truncated variants of *dMarf1* transgenes lacking either the N-terminal region, RRM domain, OST domain, C-terminal region, or 47 C-terminal amino acid residues, were generated and expressed in female germline cells to examine the role of the conserved domains (S7 Fig; Fig 4). Similar to the full-length Myc-dMarf1, all truncated dMarf1 proteins except Myc-dMarf1-ΔN lacking 252 N-terminal residues were localized to the nurse cell nucleus. Myc-dMarf1-ΔN showed faint staining in the nurse cell nuclei, suggesting that the N-terminal residues are required for nuclear localization. Although other truncated proteins predominantly accumulated in nurse cell nucleus and ooplasm, none including Myc-dMarf1-ΔN rescued the hatching rate of eggs (S1 Table). Even Myc-dMarf1-ΔC47 lacking 47 C-terminal residues was unable to rescue the hatching rate, indicating that this small but conserved region is important for dMarf1 function in insects.

### Dhd thioredoxin required for pronuclei fusion is downregulated in *dMarf1* mutant ovaries

**Fig 5:**
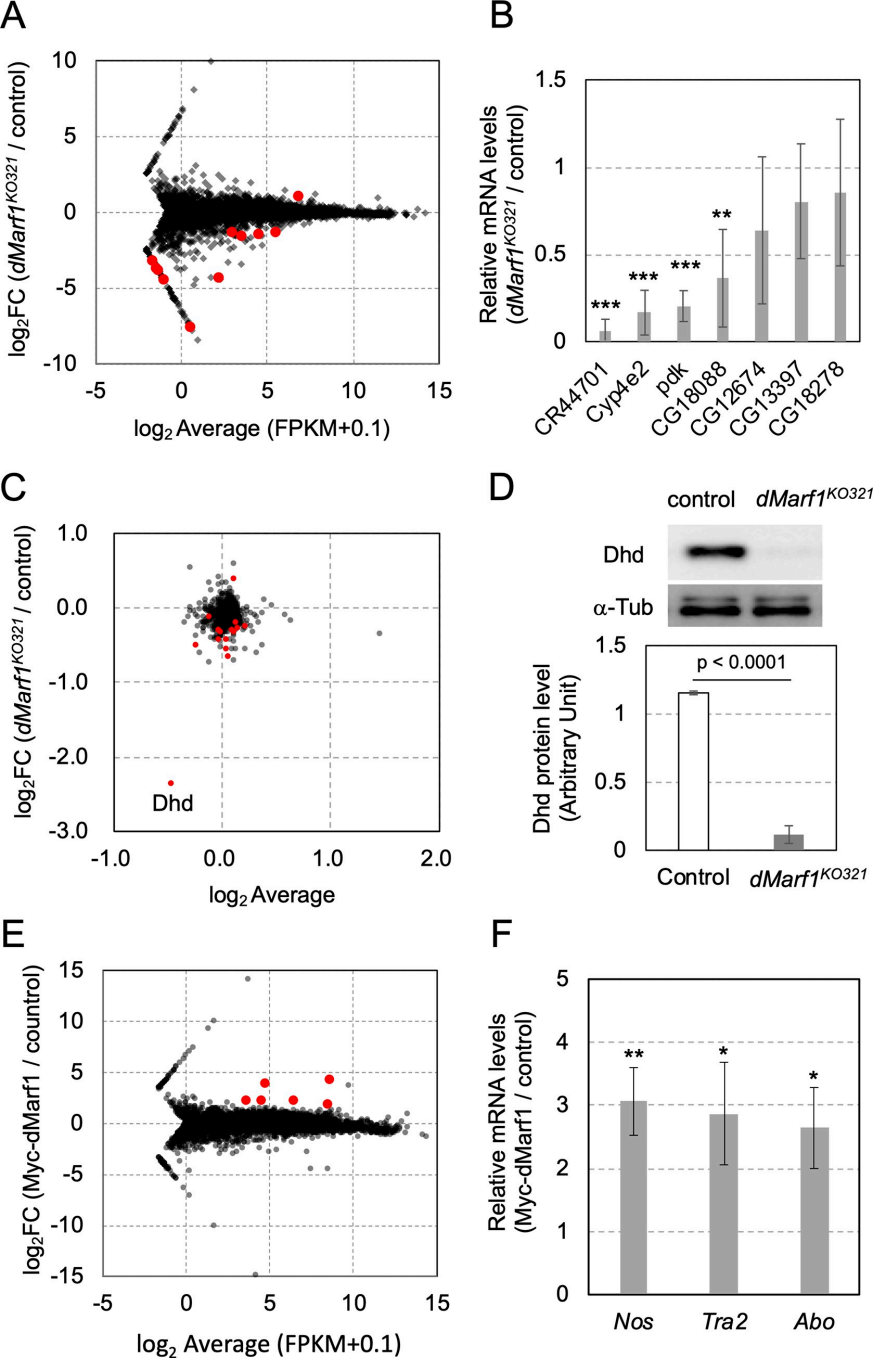
Global analysis of gene expression in *dMarf1*^*KO321*^ ovaries and dMarf1-bound mRNA in Myc-dMarf1 expressing ovaries. (A) MA plot for mRNA-seq analysis. The average RPKM (Reads Per kilobase per Million reads) values for each gene in *dMarf1*^*KO321*^ and the control ovaries are plotted on the X-axis. The log2-fold change in the expression of each gene in *dMarf1*^*KO321*^ compared to that in the control ovaries are plotted on the Y-axis. Red dots indicate genes whose expressions are significantly different between *dMarf1*^*KO321*^ mutant and the control ovaries (p-value < 0.0001). (B) qRT-PCR validation of differentially expressed genes between the control and *dMarf1*^*KO321*^ ovaries. Values represent the mean and standard deviation of three replicates. The significant differences of student’s t-test are indicated by ** (p-value < 0.01) or *** (p-value < 0.001). (C) MA plot for quantitative proteome analysis of stage 14 egg chambers. The average log2 values in *dMarf1*^*KO321*^ mutant and the control ovaries for each detected protein are plotted on the X-axis. The log2-fold changes of each detected protein in *dMarf1*^*KO321*^ mutant compared to that in the control ovaries are plotted on the Y-axis. Red dots indicate proteins that show significantly different expression levels between the mutant and the control ovaries (p-value < 0.05). (D) Western blot showing Dhd expression in stage 14 *dMarf1*^*KO321*^ egg chambers and control (upper panel). The band intensity represents the mean of three biological replicates. The mean and standard deviation are shown in the lower panel (p < 0.0001; Student’s t-test). α-Tubulin was used as the loading control. (E) MA plot for dMarf1-bound RNA. RNAs bound to Myc-dMarf1 were immunoprecipitated and analyzed by next-generation sequencing. The average RPKM values of each gene whose transcript bound to dMarf1 and the control beads are plotted on the X-axis. The log2-fold change of each gene whose transcript bound to dMarf1 compared to control are plotted on the Y-axis. Red dots indicate genes whose transcripts are significantly enriched with dMarf1 (p-value < 0.005, and log2-fold change > 1.8). (F) qRT-PCR validation of enriched mRNAs in Myc-dMarf1 between the control and *dMarf1*^*KO321*^ ovaries. Values represent the mean and standard deviation of three replicates. The significant differences of student’s t-test are indicated by * (p-value < 0.05) or ** (p-value < 0.01).

Consistent with the finding that dMarf1 is expressed in the late stages of oogenesis (Fig 1C), meiotic progression beyond metaphase I—the second arrest point—was not observed in the absence of dMarf1 (Fig 3), suggesting that dMarf1 is required for proper oocyte maturation. In an attempt to obtain candidate genes those expressions were affected by the loss of dMarf1, we first conducted the next-generation sequencing (mRNA-seq) of each one RNA library from *dMarf1*^*KO321*^ mutant and the heterozygous control ovaries. The mRNA expression levels of 10 genes were significantly decreased or increased in *dMarf1*^*KO321*^ ovaries compared to those in the control (p-value < 0.0001, Fig 5A, S2 Table). We further validated these changes with three biological replicated qRT-PCR, and identified four genes (*CR44701*, *Cyp4e2*, *pdk*, and *CG18088*) whose expression was downregulated (Fig 5B). *pdk* encodes pyruvate dehydrogenase kinase, a negative regulator of the pyruvate dehydrogenase complex. Pyruvate metabolism regulates the balance between glucose uptake and glycogen storage in oocytes during maturation [25]. Thus, a decrease in Pdk levels may affect the metabolome of *dMarf1* mutant oocytes and perturb embryogenesis.

Other genes whose mRNA expression was altered in *dMarf1* mutant ovaries were not related to meiosis. We also examined defects in protein expression of stage 14 *dMarf1* mutant egg chambers. Of the 700 proteins that were detected, Dhd expression was significantly reduced to 20% in *dMarf1*^*KO321*^ compared to that in the control (Fig 5C; S3 Table). Western blotting confirmed that only 10% of Dhd was expressed in the *dMarf1* mutant relative to that in the control (Fig 5D). In *dMarf1*^*KO321*^ ovaries, however, the mRNA expression of Dhd was not affected despite a significant reduction in its protein expression (S8A Fig). This result suggests that Dhd expression is post-transcriptionally regulated. Dhd is a thioredoxin whose expression dramatically increases during oocyte maturation [26]. Unlike the *dMarf1*^*KO321*^ mutant, *dhd* mutant females exhibit defects in unpacking paternal chromosomes before the fusion of pronuclei [27], suggesting that the reduction in Dhd expression is not the causative factor for meiotic defect in *dMarf1*^*KO321*^ oocytes. Our results suggest that *dMarf1* regulates not only meiosis but also subsequent steps including pro-nuclear fusion at the onset of embryogenesis.

### dMarf1 binds to *nos* mRNA to regulate its expression

It is known that dMarf1 harbors a conserved RRM domain; hence, we screened for RNA molecules bound by dMarf1 to identify its direct effectors. Myc-dMarf1-FL protein expressed in the germline cells was immunoprecipitated. To obtain the potential genes whose mRNAs bound by dMarf1 protein, the co-precipitated RNAs were analyzed by next-generation sequencing. Six different RNAs were enriched in the immunoprecipitated fraction (Fig 5E and S4 Table; log2 fold change > 1.5 and p-value < 0.01). Of these, the enrichment of *nanos* (*nos*), *transformer 2* (*tra2*), and *abnormal oocyte* (*abo*) mRNAs in the Myc-dMarf1 bound fraction was further confirmed by qRT-PCR (Fig 5F). *tra2* encodes an RNA-binding protein that functions together with *transformer* (*tra*) to promote sex-specific splicing of *doublesex* (*dsx*) mRNA [28]. *abo* is a negative regulator of histone gene transcription [29]. Although both *tra2* and *abo* are not directly involved in meiosis or oocyte maturation, dMarf1 is suggested to function by binding with *tra2* and *abo* mRNAs, which will be discussed later.

**Fig 6:**
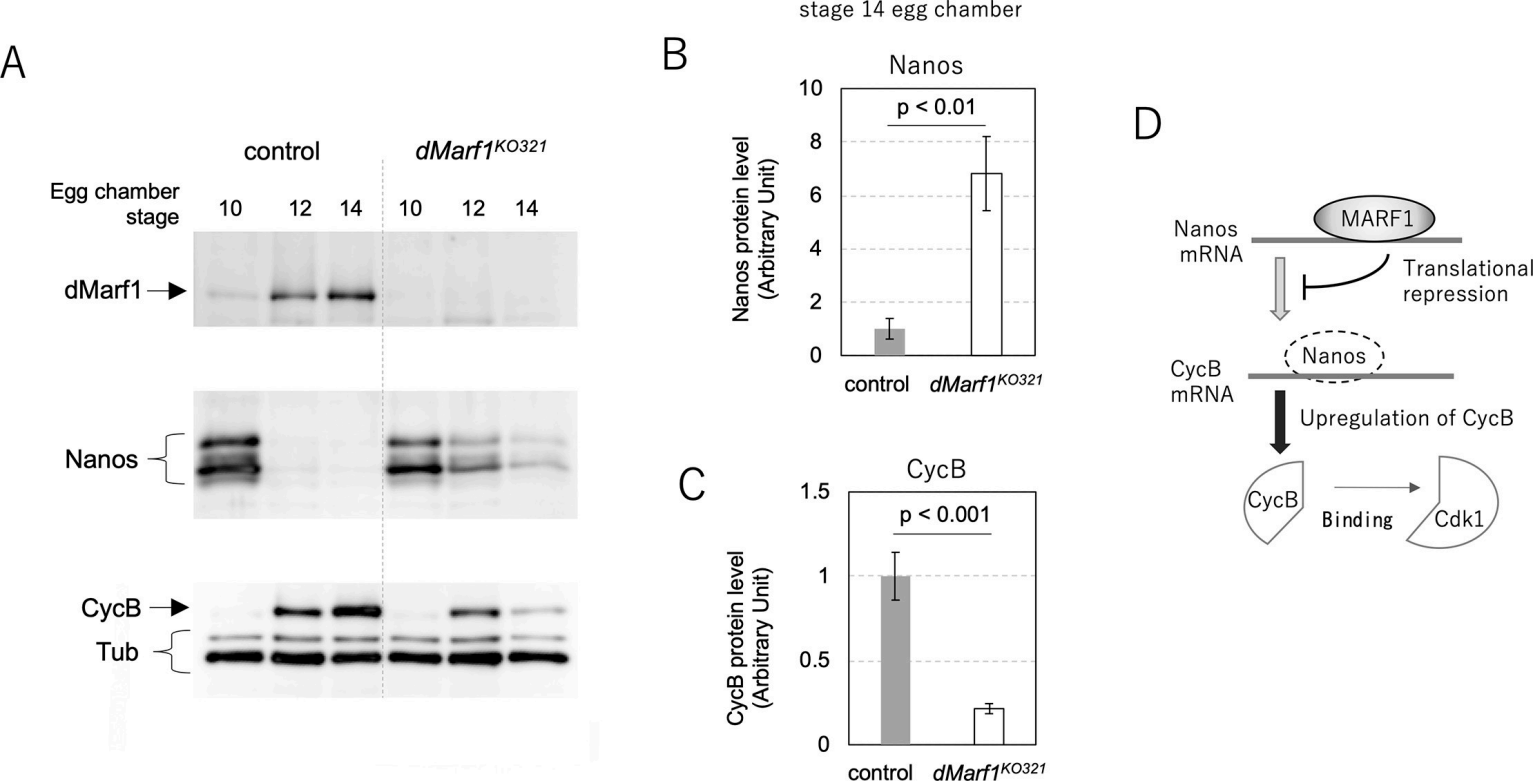
*dMarf1* translationally represses Nos in the late stages of oogenesis. (A) Western blot showing protein expression at each stage of oogenesis. Upper panel, dMarf1; middle panel, Nos; bottom panel, Cyclin B, and the loading control, α-tubulin. (B) Quantitative analysis of Nos protein expression in stage 14 egg chambers. The mean and standard deviation of Nos expression are shown in terms of arbitrary unit (p < 0.01; Student’s t-test). (C) Quantitative analysis of CycB protein expression in stage 14 egg chambers. The mean and standard deviation of CycB expression are shown in terms of arbitrary unit (p < 0.001; Student’s t-test). (D) Schematic model of dMarf1 function. In wild type females, dMarf1 coordinates the late stages of oogenesis. dMarf1 binds to *nos* mRNA and inhibits its protein translation. Consequently, CycB expression is upregulated at the onset of oocyte maturation, which results in the formation of the MPF complex to promote meiosis. In the absence of dMarf1, Nos is expressed until stage14 and CycB is downregulated, which in turn reduces MPF activity.

Another dMarf1-bound mRNA, *nos*, encodes a translational repressor of *cycB*, the protein of which complexes with Cdk1 to form maturation promoting factor (MPF) [30]. To examine whether dMarf1 regulates *nos* expression by binding to its RNA, we analyzed Nos protein expression in the late stages of oogenesis. Consistent with the results of the previous study, Nos was highly expressed in stage 10 egg chambers and its expression rapidly decreased in the later stages [31] (Fig 6A, middle panel). Nos protein levels in stage 10 *dMarf1*^*KO321*^ egg chambers were comparable to that in stage 10 control egg chambers (Fig 6A). However, Nos expression decreased gradually, but remained detectable even in the stage 14 egg chambers; Nos expression in stage 14 *dMarf1*^*KO321*^ egg chambers was approximately 7-fold higher than that in the corresponding control egg chambers (Fig 6B). By contrast, the mRNA expression of *nos* in stage 14 *dMarf1*^*KO321*^ egg chambers remained unchanged (S8A Fig). These results suggest that dMarf1 can function as a translational repressor of *nos* mRNA, although the possibility of delayed or aberrant degradation of Nos in *dMarf1*^*KO321*^ ovaries cannot be ruled out.

### CycB protein expression is reduced in *dMarf1* mutant ovaries

Because Nos, together with Pumilio, is known to translationally repress CycB in embryos [32], we examined CycB expression in stage 14 *dMarf1*^*KO321*^ egg chambers where Nos protein expression is remarkably upregulated. In the control ovaries, CycB accumulation was observed in stage 14 egg chambers along with the downregulation of Nos. However, CycB protein expression in stage 14 egg chambers of *dMarf1*^*KO321*^ was five-fold lower than that in the control (Fig 6A, bottom panel, and 6C). Moreover, *cycB* mRNA expression was not significantly affected (S8 Fig). Our results suggest that Nos functions as a translational repressor of *cycB* not only during embryonic development but also oocyte maturation. The downregulation of CycB expression in the late stages of oogenesis in *dMarf1*^*KO321*^ females reduces MPF activity, and can potentially inhibit meiotic progression.

CycB3 is another cyclin that is required for anaphase progression in meiosis I and II. [4]. CycB3 expression was only slightly decreased in *dMarf1*^*KO321*^ (S9B Fig), confirming that CycB, rather than CycB3, is a major effector of dMarf1 regulation. MPF activity is also required to induce the expression of PNG kinase complex components (Png/Plu/Gnu) that are important for the activation of stage 14 oocytes [33]. Consistent with the reduction of MPF activity in *dMarf1*^*KO321*^, only the protein expression but not the mRNA expression of Gnu was also reduced (S9B Fig, S8A Fig). Taken together, our results suggest that dMarf1 functions to coordinate oocyte maturation, at least in part, through regulation of the MPF component, CycB, via Nos-induced translational repression.

## Discussion

### dMarf1 may regulate Nos during late oogenesis

Nos is an evolutionary conserved protein that play important roles in early embryogenesis, formation of primordial germ cells and maintenance of germline stem cells (GSCs) [31,34,35]. However, the function of Nos during late oogenesis has not been described yet. Nos protein expression reaches the maximum around stage 10 of oogenesis and immediately reduces thereafter [31] (Fig 6A). This suggests that Nos expression is tightly regulated during late oogenesis. Our results indicate that dMarf1 regulates Nos expression by inhibiting its translation in late oogenesis. In the absence of dMarf1 expression during early-to-mid oogenesis, Nos might repress *cycB* to prevent the premature release of meiotic arrest until stage 10 of oogenesis. Our results suggest that dMarf1 may coordinate oocyte maturation during late oogenesis in *Drosophila* (Fig 6D); moreover, dMarf1 is dominantly expressed after stage 10, and binds to *nos* mRNA to repress its translation and reduce its expression. Consequently, Nos expression almost disappears at stages 12–14. Reduced expression of Nos induces the release of *cycB* mRNA from repression and promotes CycB translation. Subsequently, CycB binds to Cdk1 to form the active MPF complex to promote meiosis. Therefore, dMarf1 plays an important role in the release of the second meiotic arrest to drive embryogenesis.

### The conserved domain and region of MARF1 protein have distinct functions

The *MARF1* gene is evolutionarily conserved in animals; most proteins of the MARF1 family contain three major domains: NYN, OST (also known as LOTUS), and RRM (S1 Fig). Although the ribonuclease activity of NYN is essential for MARF1 function in mouse [7], it is not required in *Drosophila* dMarf1. The OST domain is present in the proteins of several species ranging from bacteria to humans [19,20]. *Drosophila melanogaster* has four members of OST domain-containing proteins: dMarf1, Oskar (Osk), Tejas (Tej), and Tapas (Tap) [13,36,37]. All of these proteins except dMarf1 contain a single OST domain, and are predominantly expressed in germline cells. Structural and biochemical studies of Osk, Tej, and Tap OST domains have revealed the ability of this domain to bind to Vasa, an RNA helicase expressed exclusively in germline cells [38]. Interestingly, the OST domain(s) of MARF1 family members is smaller than those of other proteins and does not bind to Vasa. Instead, a recent study reported that dMarf1 could bind to CCR4-Not deadenylase complex via the OST domain(s); however, the importance and cooperation between multiple OST domains remains elusive [8]. In addition to these two domains, the MARF1 family members contain one or two RRM domains. In this study, we showed that dMarf1 translationally repressed *nos* mRNA. *nos* may not be the only target of dMarf1; other mRNAs such as *tra2* and *abo* can also be targeted by dMarf1, although the biological significance of these interactions remains unclear. Zhu et al. have recently reported that dMarf1 can bind to *cyclin A* mRNA via its RRM domain [8]. However, our RNA-IP analysis did not detect *cyclin A* in the dMarf1-bound mRNA fraction (S4 Table). Further studies of the molecular mechanism underlying the specificity of dMarf1 RNA binding will reveal the range of mRNA regulation during oocyte maturation.

The C-terminal region of the MARF1 family members is highly conserved among higher animals, except insects (S3 and S4 Figs), despite not forming any secondary structure. The C-terminal region of human MARF1 has been shown to directly interact with the decapping complex component Ge-1 [21]; however, this interaction was not observed in *Drosophila* [8]. Moreover, the C-terminal region of the MARF1 family members is conserved among different insect species, but different from that of higher animals (S4 Fig), indicating that it may bind to a unique partner in insects. This hypothesis was supported by our results showing that transgenic dMarf1 mutant lacking 47 C-terminal residues (ΔC47) could not rescue *dMarf1*^*KO321*^ mutant phenotype (S1 Table).

### Both *Drosophila* and mouse *Marf1* are involved in RNA regulation, but their modes of function are different

In addition to binding to the RNA decapping complex subunit [21,39], human MARF1 can localize to processing body (P-body), which is often related to translational repression and mRNA decay [40]. Mouse MARF1 has also been shown to degrade target mRNA via its NYN domain, which is absent in *Drosophila* dMarf1 [7]. Transcriptome analysis of mouse MARF1 mutant oocytes revealed that 1,470 transcripts were upregulated in the steady state, whereas 103 transcripts were downregulated [7], indicating a global impact on RNA homeostasis in the mutant oocytes. By contrast, the expression of a few RNAs was downregulated and *dp1* mRNA expression was upregulated in *Drosophila dMarf1* mutant ovaries (Fig 5A, S2 Table). These results suggest that mammalian MARF1 may regulate the global transcriptome predominantly by degradation, while dMarf1 represses translation of target proteins such as CycB and CycA by modulating Nos/Pumilio and the CCR4-NOT deadenylase complex, respectively [37].

In addition to *nos* mRNA, dMarf1 can bind to other mRNAs, including *tra2* and *abo*. The mRNA expression of *tra2* was not significantly affected in stage 14 *dMarf1*^*KO312*^ egg chambers (S8A Fig), suggesting that dMarf1 post-transcriptionally regulates *tra2* expression. *abo* is a negative regulator of histone gene expression and its expression is downregulated in mature oocytes to produce more histones [41]. The expression of *abo* mRNA was upregulated by approximately three-fold in stage 14 *dMarf1*^*KO321*^ egg chambers (S8A Fig). This may result in the overexpression of Abo protein in dMarf1 mutant oocytes, which in turn causes the downregulation of histone proteins that are required for embryogenesis.

The *Ppp2cb* gene encodes a protein phosphatase that is involved in cell cycle regulation. *Ppp2cb* has been previously reported as a major downstream effector of mouse MARF1 [42]. The high expression of Ppp2CB phosphatase in the MARF1 mutant ovaries of mouse can disrupt meiosis. However, the expression of *mts*, a *Drosophila* homolog of *Ppp2cb*, was not affected in *dMarf1* mutant ovaries (S8A Fig), suggesting that the signaling pathway for the activation of the M phase promoting factor, CycB/Cdk1, is not conserved between mouse and *Drosophila*. Although the direct activation of MPF in mouse MARF1 mutant oocytes by the inhibition of *Ppp2cb* rescued meiotic defect, embryogenesis of mutant oocytes was affected [42], suggesting that *Ppp2cb* may have additional functions in addition to MPF activation. Similarly, *dMarf1*^*KO321*^ ovaries exhibited not only meiotic defects, but also translationally downregulated some proteins required for embryogenesis, such as Dhd and Gnu (Fig 5D, S8A Fig, and S9B Fig). In conclusion, MARF1 may trigger oocyte maturation and coordinate multiple events during late oogenesis and fertilization.

## Supporting information

S1 Fig Members of the MARF1 family are conserved in animals. (A) Domain structures and multiple sequence alignments of the MARF1 family members. The polypeptide length is shown on the right. The domain structure is accessed from the NCBI. The NYN, RRM, and OST domains are denoted by blue, orange, and green boxes, respectively. (B) The phylogenic tree of MARF1 family members was generated using neighbor-joining method in CLC Genomics Workbench (Qiagen). The distance scale is also indicated. The NCBI reference sequences of the proteins are as follows: NP_724394 for *Drosophila melanogaster*, NP_055462 for *Homo sapiens*, NP_001074623 for *Mus musculus*, XP_021332636 for *Danio rerio* (zebrafish), NP_001119538 for *Xenopus tropicalis* (western clawed frog), XP_020900751 for *Exaiptasia pallida* (sea anemone), and XP_011405091 for *Amphimedon queenslandica* (sponge).
(PDF)

S2 Fig Secondary structure prediction of dMarf1. Secondary structure of dMarf1 polypeptide was predicted using the PSIPRED method. The vertical blue bar represents prediction confidence for each residue, and alpha helices and beta sheets are shown in pink barrels and yellow arrows, respectively.
(PDF)

S3 Fig Multiple sequence alignment of MARF1 C-terminal region. The C-terminal regions of MARF1 in various animal species are aligned. The bar under the alignment indicates sequence conservation (highly conserved in red, less conserved in blue). The alignment and schematic representation were designed in CLC Genomics Workbench (Qiagen).
(PDF)

S4 Fig Multiple sequence alignment of insect MARF1 family members. The multiple sequence alignment for the C-terminal region of the insect MARF1 family members is shown. The bar under the alignment indicates sequence conservation at each residue (highly conserved in red, less conserved in black). The alignment and schematic representation were designed in CLC Genomics Workbench (Qiagen). The NCBI reference sequences of the proteins are as follows: Q7KWG9_*Drosophila melanogaster* (DROME), A0A0J9R6W8_*Drosophila simulans* (DROSI), B3NKL7_*Drosophila erecta* (DROER), B4IT21_*Drosophila yakuba* (DROYA), B4LQH5_*Drosophila virilism* (DROVI), B3MX08_*Drosophila ananassae* (DROAN), A0A1A9UH46_*Glossina austeni* (GLOAU), A0A1A9XT70_*Glossina fuscipes fuscipes* (GLOFF), A0A1A9W5J8_*Glossina brevipalipis* (9MUSC), and A0A0L0C5F0_*Lucilia cuprina* (LUCCU).
(PDF)

S5 Fig *dMarf1* functions in germline cells for female fertility. (A) *dMarf1*^*KO321*^ homozygous mutant males expressing a loss-of-function allele did not show significant defects in fertility. (B) *dMarf1*^*KO321*^ homozygous females laid eggs, but they did not hatch. (C-D) The expression of *dMarf1* was knocked down using an shRNA under three different drivers: germline drivers, *NGT40*; *nosGal4* and *Matα*, and a somatic driver, *tj-Gal4*. The number of eggs laid by *dMarf1-KD* females within 24 h is plotted in (C). The hatching rate of eggs laid by *dMarf1-KD* females within 24 h is plotted in (D). The p-value of the student’s t-test is shown in each graph.
(PDF)

S6 Fig Transgenic Myc-dMarf1 can rescue meiotic defects in dMarf1 mutant females. (A) The hatching rates of eggs laid by *dMarf1* mutant females and rescue females expressing full-length Myc-dMarf1 in germline cells within 24 h. The p-value of the student’s t-test is shown in graph. (B) Embryos from the control, mutant, and rescue females are stained with DAPI (blue), anti-*α*-tubulin (green), and anti-phospho-histone H3 (red). Scale bar, 5 μm (C) The progression of embryo development 0–6 h after laying eggs for control, mutant, and rescue females. The developmental stage (before or after blastoderm) was analyzed by the assessing the distribution of chromosomes in the DAPI-stained embryos.
(PDF)

S7 Fig Schematic representation of full-length dMarf1 and its deletion variants. The dotted parts represent the truncated regions. RRM and OST denote RNA recognition motif and OST/Lotus domain, respectively.
(PDF)

S8 Fig Quantification of mRNA expression in stage 14 egg chambers and Myc-dMarf1 bound fraction. (A) mRNA expression in stage 14 egg chambers was quantified using qRT-PCR. The fold enrichment of *dMarf1*^*KO321*^ compared to the control is plotted. The p-values of student’s t-test are indicated by ns (p-value > 0.05) or * (p-value < 0.05). (B) mRNA bound to Myc-dMarf1 was quantified using qRT-PCR. The fold enrichment compared to the control is plotted. The p-values of student’s t-test are indicated by ns (p-value > 0.05) or * (p-value < 0.05).
(PDF)

S9 Fig Quantification of protein expression during late oogenesis. (A) Western blot showing protein expression at each stage of egg chambers in the heterozygous control and *dMarf1*^*KO321*^ females. (B) Quantitative analysis of CycB3 and Gnu protein expression in stage 14 egg chambers. The mean and standard deviation values are shown in terms of arbitrary unit. The p-value of the student’s t-test is also shown in the graph.
(PDF)

S1 Table Hatching ratio of embryos expressing truncated dMarf1. (XLSX)

S2 Table Identification of genes by mRNA-seq analysis. (XLSX)

S3 Table Identification of proteins by quantitative mass analysis. (XLSX)

S4 Table Identification of genes by RIP-seq analysis. (XLSX)

S5 Table Primers used in this study. (XLSX)

S1 Protocol The detail descriptions about generating dMarf1 mutant fly. (DOCX)

S1 File Raw images of western blotting analyses. (PDF)
