# Supplementary figures and images for: Drosophila MARF1 ensures proper oocyte maturation by regulating nanos expression
Source: PLoS One. 2020 Apr 3;15(4):e0231114. doi: 10.1371/journal.pone.0231114 (PMC7122799; doi:10.1371/journal.pone.0231114)

# Supplementary figure 1

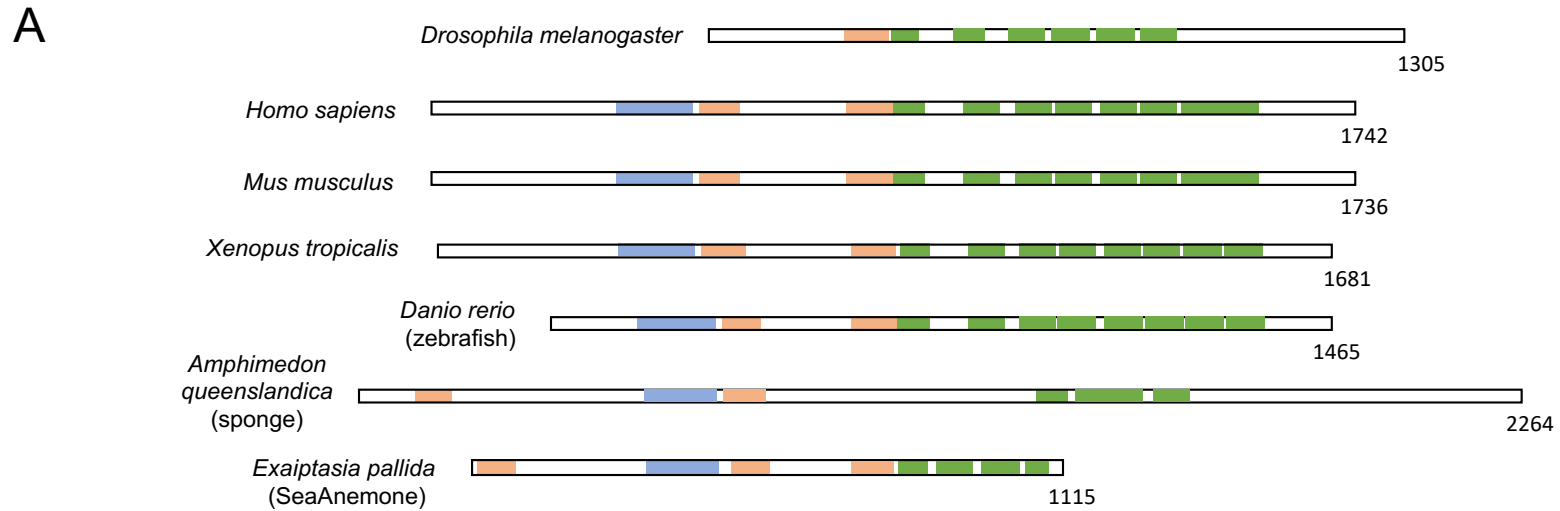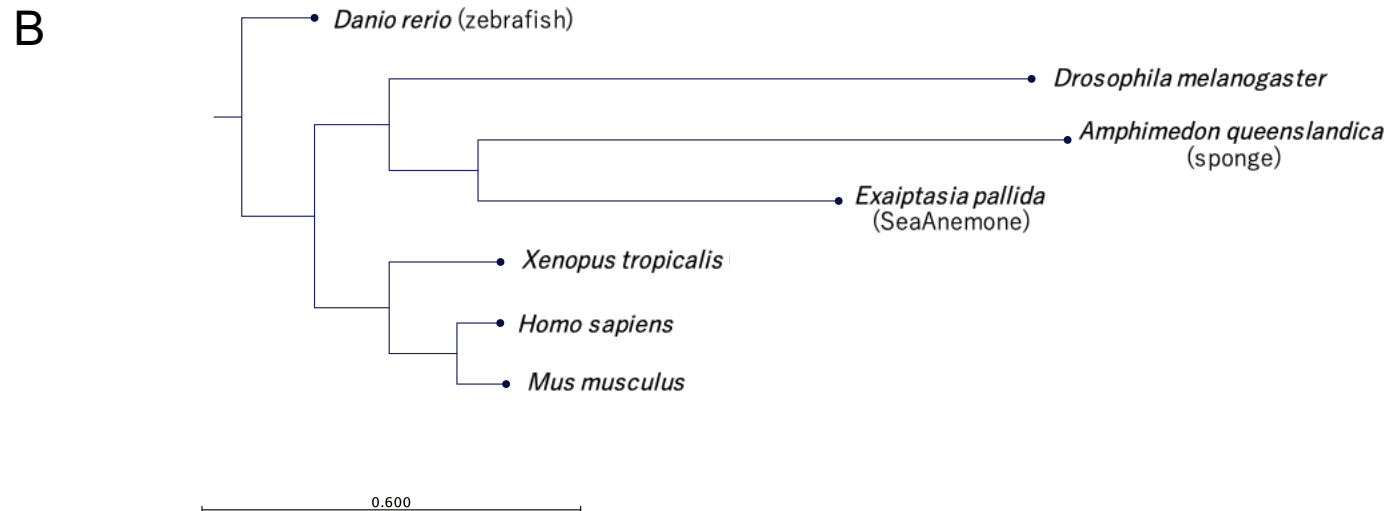

Supplement: S1 Fig — (A) Domain structures and multiple sequence alignments of the MARF1 family members. The polypeptide length is shown on the right. The domain structure is accessed from the NCBI. The NYN, RRM, and OST domains are denoted by blue, orange, and green boxes, respectively. (B) The phylogenic tree of MARF1 family members was generated using neighbor-joining method in CLC Genomics Workbench (Qiagen). The distance scale is also indicated. The NCBI reference sequences of the proteins are as follows: NP_724394 for Drosophila melanogaster, NP_055462 for Homo sapiens, NP_001074623 for Mus musculus, XP_021332636 for Danio rerio (zebrafish), NP_001119538 for Xenopus tropicalis (western clawed frog), XP_020900751 for Exaiptasia pallida (sea anemone), and XP_011405091 for Amphimedon queenslandica (sponge). (PDF) [file pone.0231114.s001.pdf]

## Supplementary figure 3

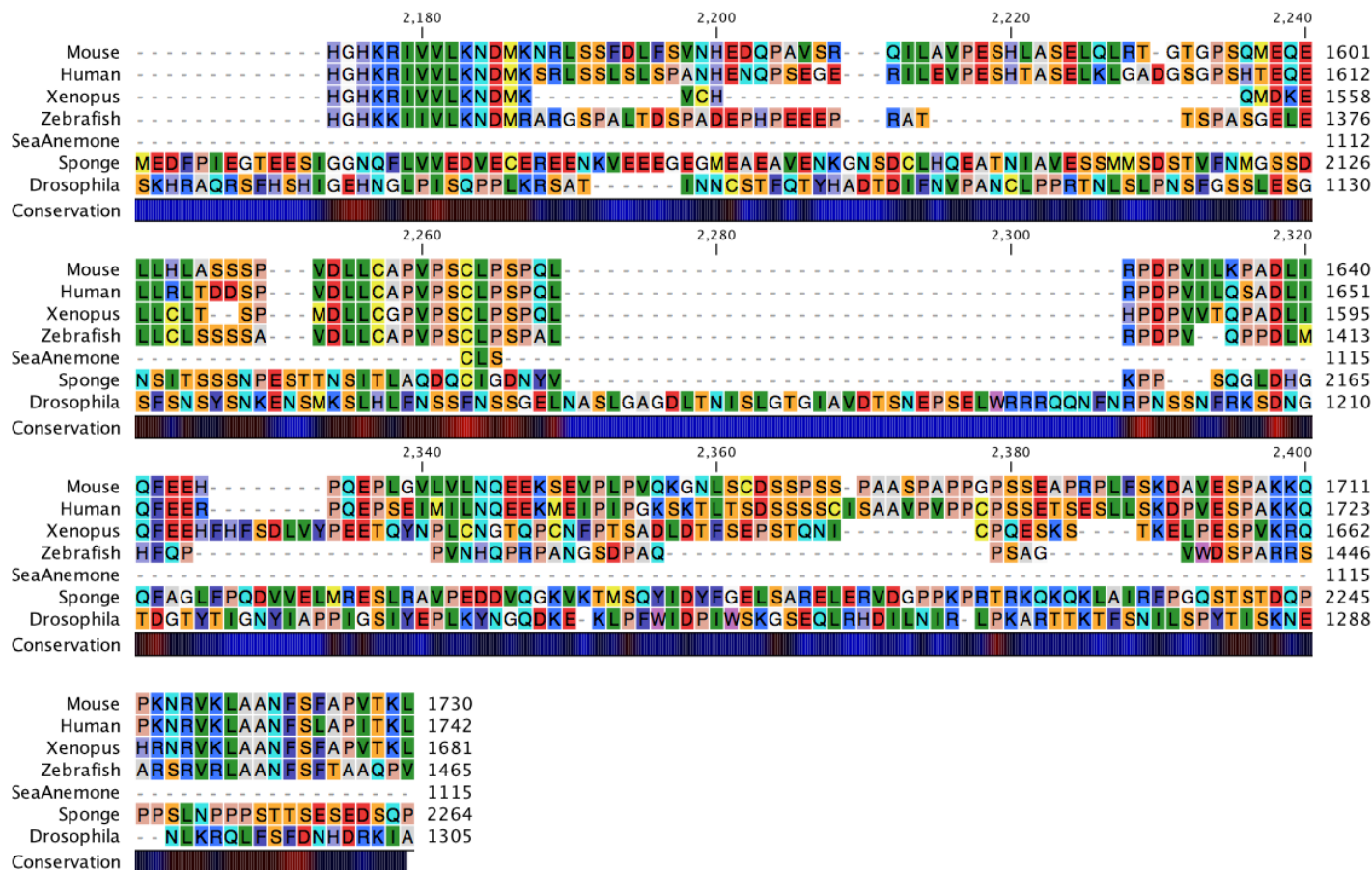

Supplement: S3 Fig — The C-terminal regions of MARF1 in various animal species are aligned. The bar under the alignment indicates sequence conservation (highly conserved in red, less conserved in blue). The alignment and schematic representation were designed in CLC Genomics Workbench (Qiagen). (PDF) [file pone.0231114.s003.pdf]

# Supplementary figure 4

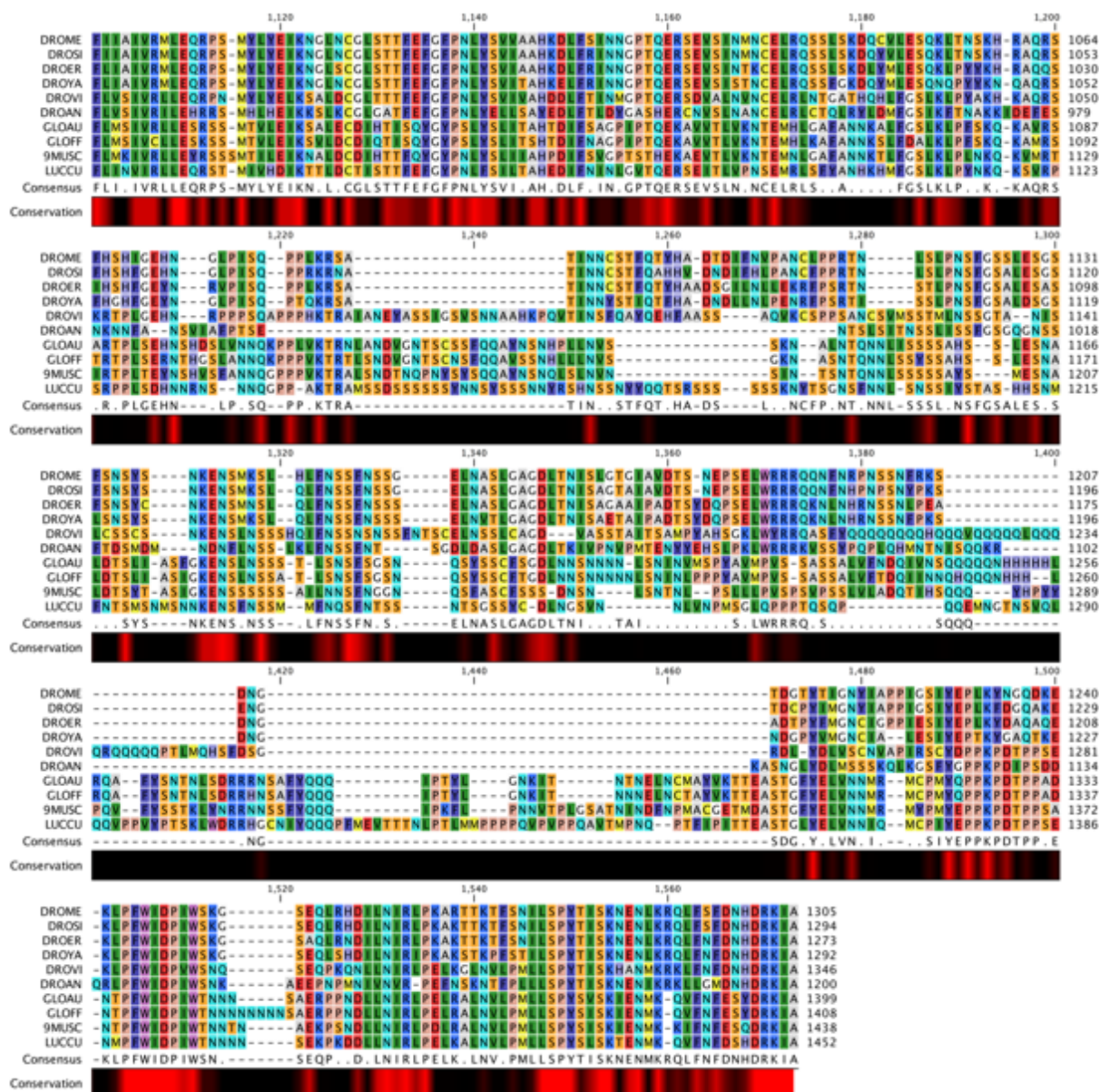

Supplement: S4 Fig — The multiple sequence alignment for the C-terminal region of the insect MARF1 family members is shown. The bar under the alignment indicates sequence conservation at each residue (highly conserved in red, less conserved in black). The alignment and schematic representation were designed in CLC Genomics Workbench (Qiagen). The NCBI reference sequences of the proteins are as follows: Q7KWG9_Drosophila melanogaster (DROME), A0A0J9R6W8_Drosophila simulans (DROSI), B3NKL7_Drosophila erecta (DROER), B4IT21_Drosophila yakuba (DROYA), B4LQH5_Drosophila virilism (DROVI), B3MX08_Drosophila ananassae (DROAN), A0A1A9UH46_Glossina austeni (GLOAU), A0A1A9XT70_Glossina fuscipes fuscipes (GLOFF), A0A1A9W5J8_Glossina brevipalipis (9MUSC), and A0A0L0C5F0_Lucilia cuprina (LUCCU). (PDF) [file pone.0231114.s004.pdf]

Supplementary figure 5

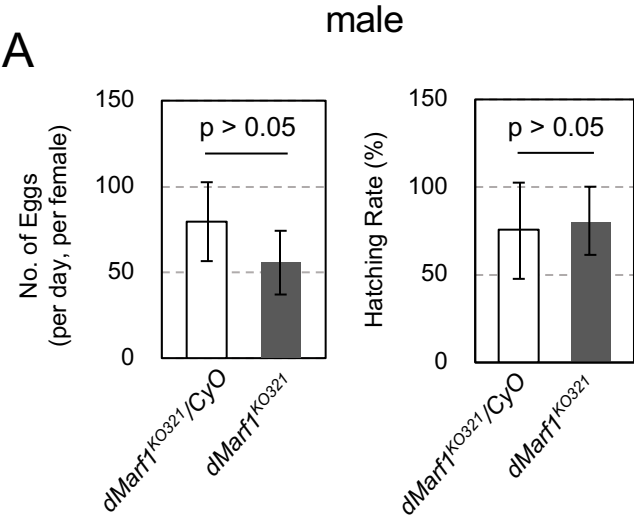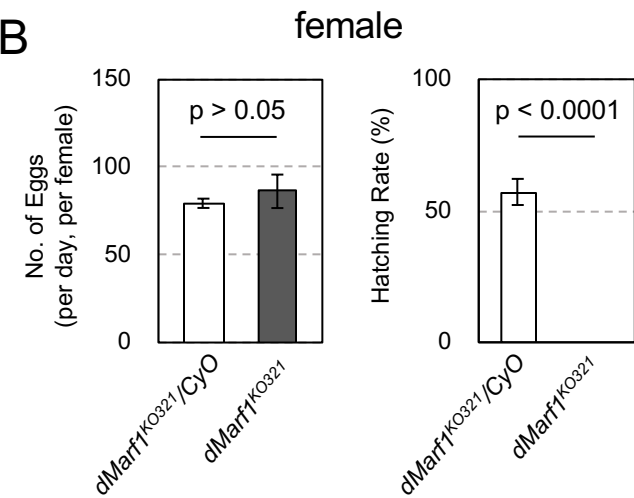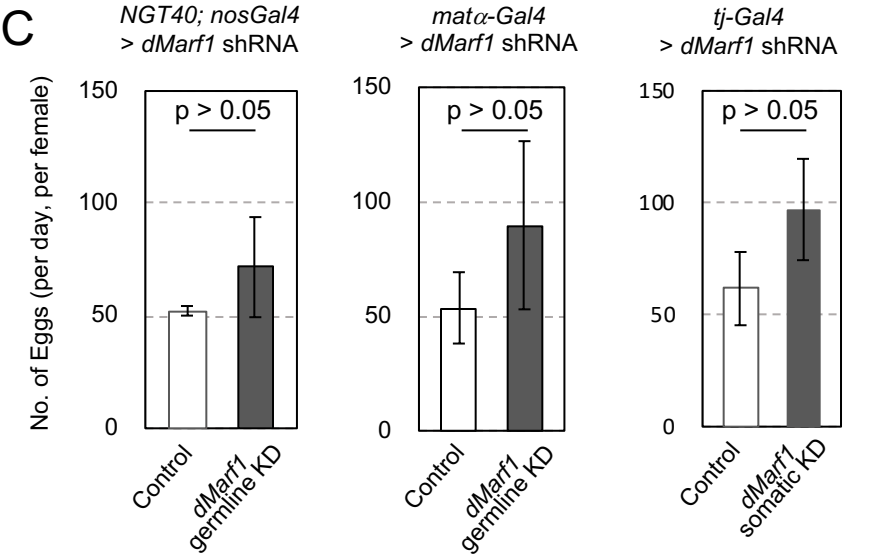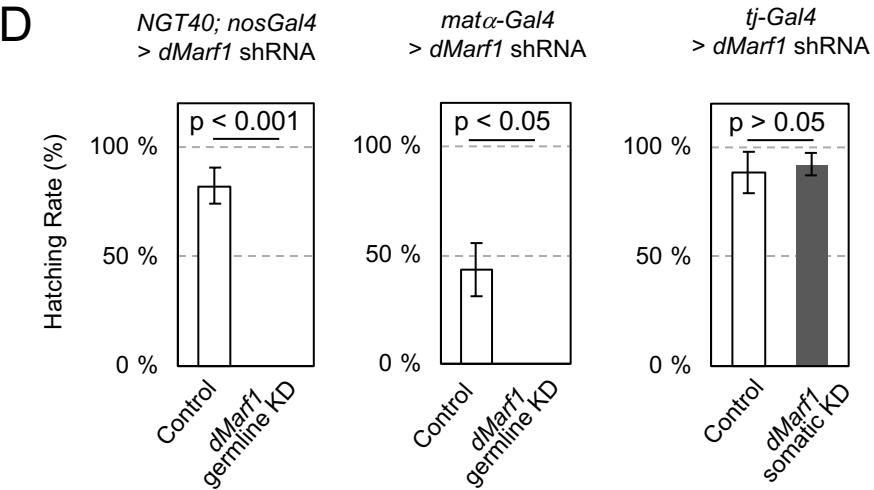

Supplement: S5 Fig — (A) dMarf1KO321 homozygous mutant males expressing a loss-of-function allele did not show significant defects in fertility. (B) dMarf1KO321 homozygous females laid eggs, but they did not hatch. (C-D) The expression of dMarf1 was knocked down using an shRNA under three different drivers: germline drivers, NGT40; nosGal4 and Matα, and a somatic driver, tj-Gal4. The number of eggs laid by dMarf1-KD females within 24 h is plotted in (C). The hatching rate of eggs laid by dMarf1-KD females within 24 h is plotted in (D). The p-value of the student’s t-test is shown in each graph. (PDF) [file pone.0231114.s005.pdf]

A

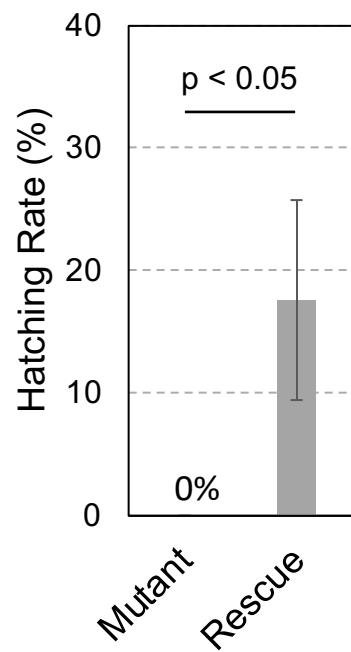

B

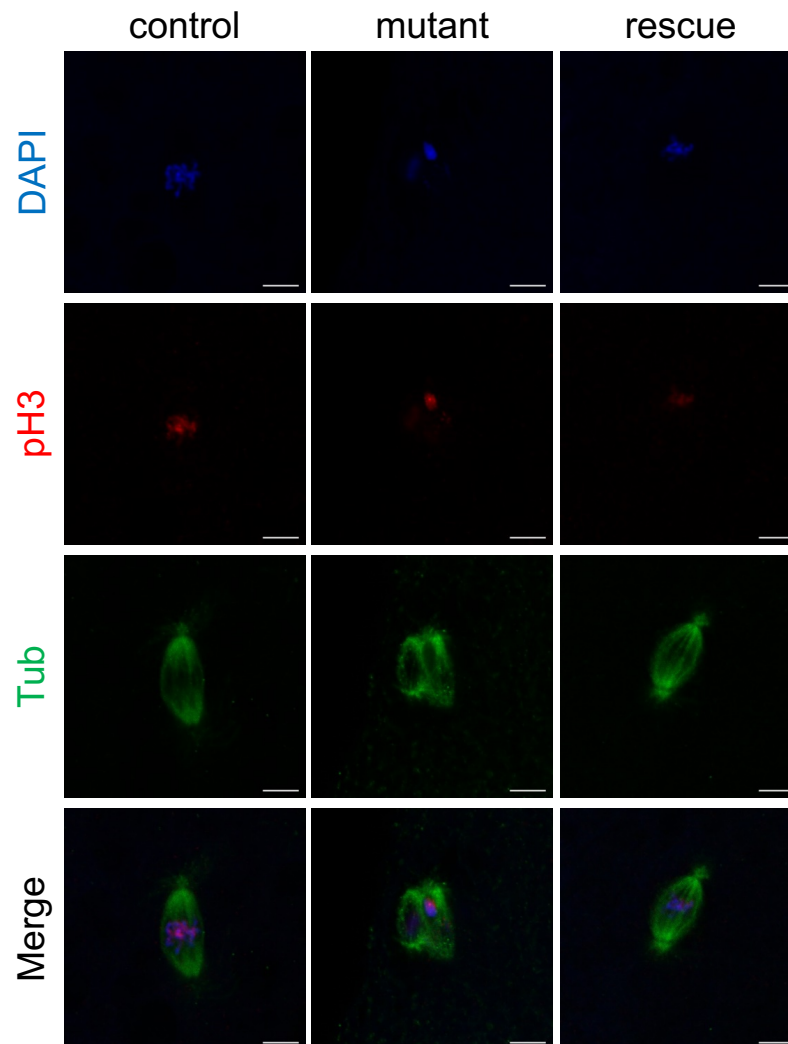

C

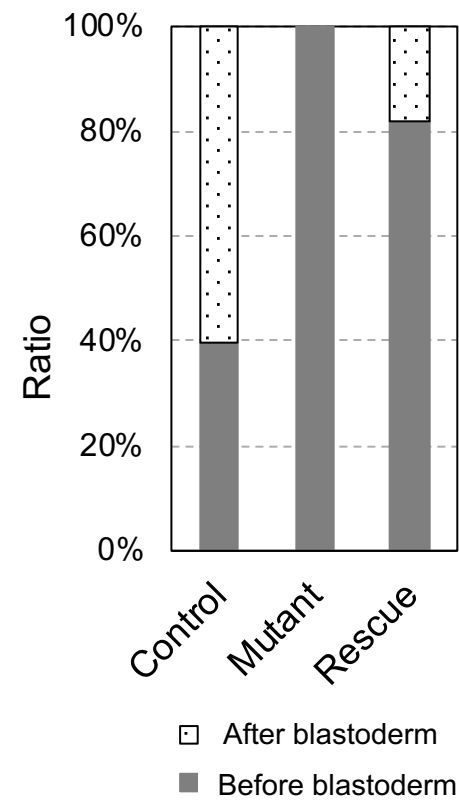

Supplement: S6 Fig — (A) The hatching rates of eggs laid by dMarf1 mutant females and rescue females expressing full-length Myc-dMarf1 in germline cells within 24 h. The p-value of the student’s t-test is shown in graph. (B) Embryos from the control, mutant, and rescue females are stained with DAPI (blue), anti-α-tubulin (green), and anti-phospho-histone H3 (red). Scale bar, 5 μm (C) The progression of embryo development 0–6 h after laying eggs for control, mutant, and rescue females. The developmental stage (before or after blastoderm) was analyzed by the assessing the distribution of chromosomes in the DAPI-stained embryos. (PDF) [file pone.0231114.s006.pdf]

Supplementary figure 7

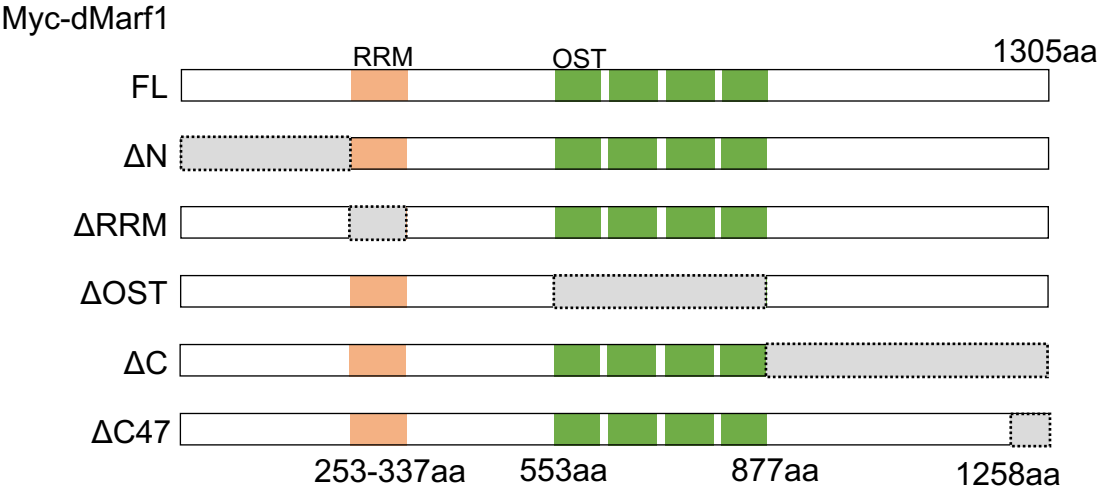

Supplement: S7 Fig — The dotted parts represent the truncated regions. RRM and OST denote RNA recognition motif and OST/Lotus domain, respectively. (PDF) [file pone.0231114.s007.pdf]

A

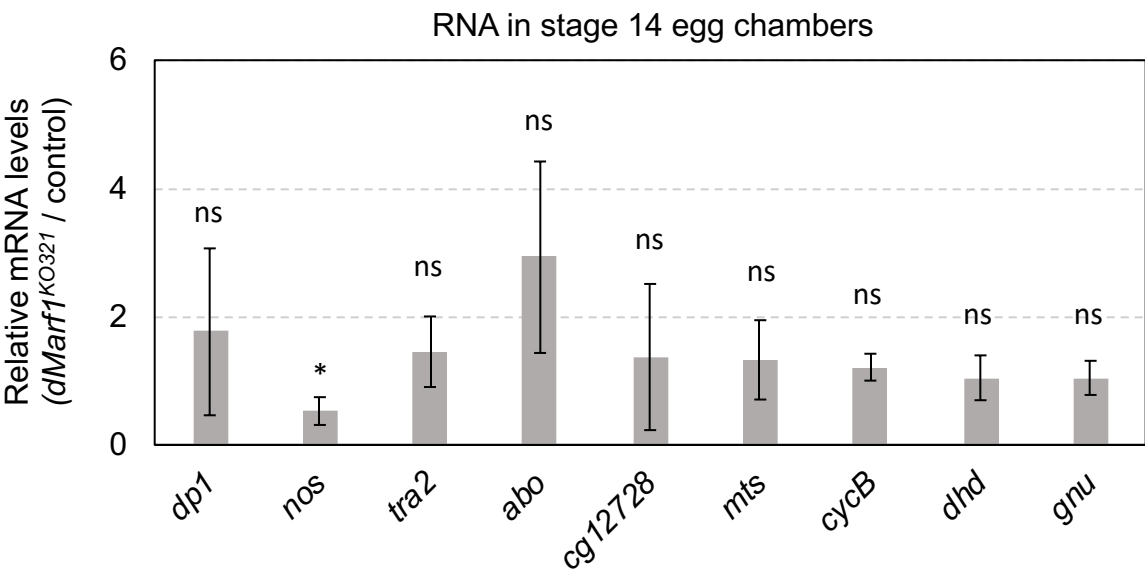

B

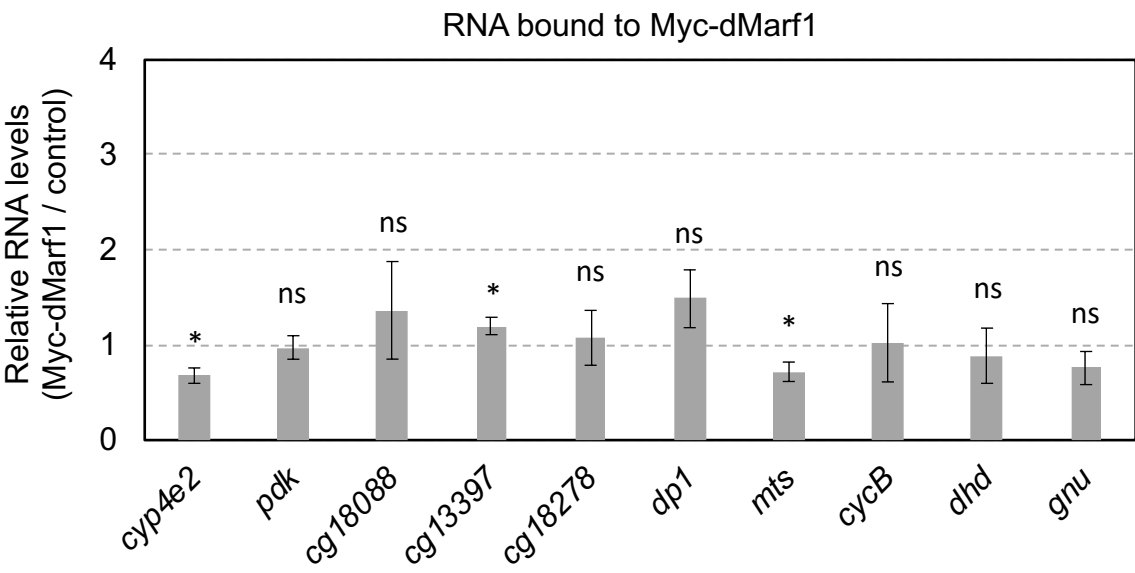

Supplement: S8 Fig — (A) mRNA expression in stage 14 egg chambers was quantified using qRT-PCR. The fold enrichment of dMarf1KO321 compared to the control is plotted. The p-values of student’s t-test are indicated by ns (p-value > 0.05) or * (p-value < 0.05). (B) mRNA bound to Myc-dMarf1 was quantified using qRT-PCR. The fold enrichment compared to the control is plotted. The p-values of student’s t-test are indicated by ns (p-value > 0.05) or * (p-value < 0.05). (PDF) [file pone.0231114.s008.pdf]

Supplementary figure 9

**A**

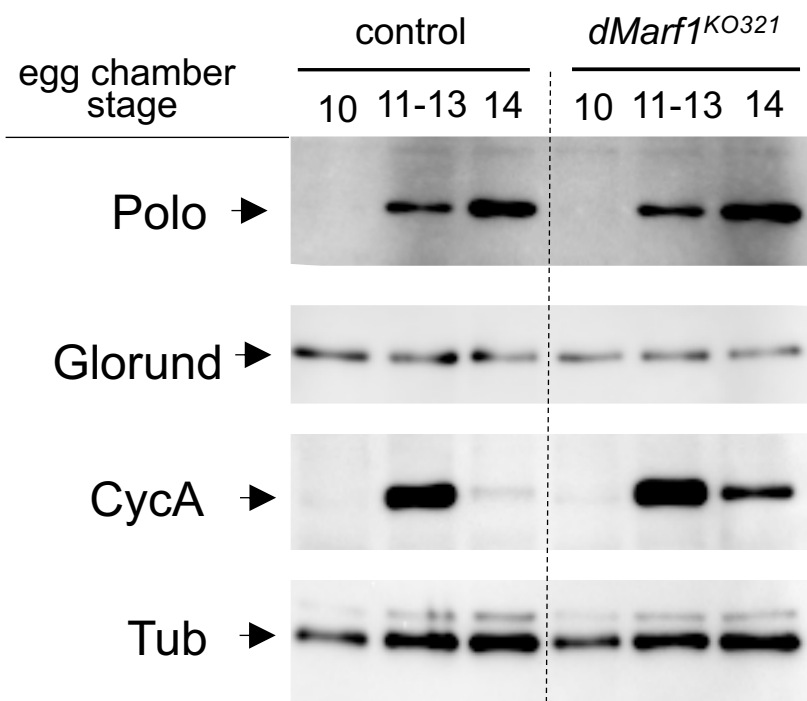

**B**

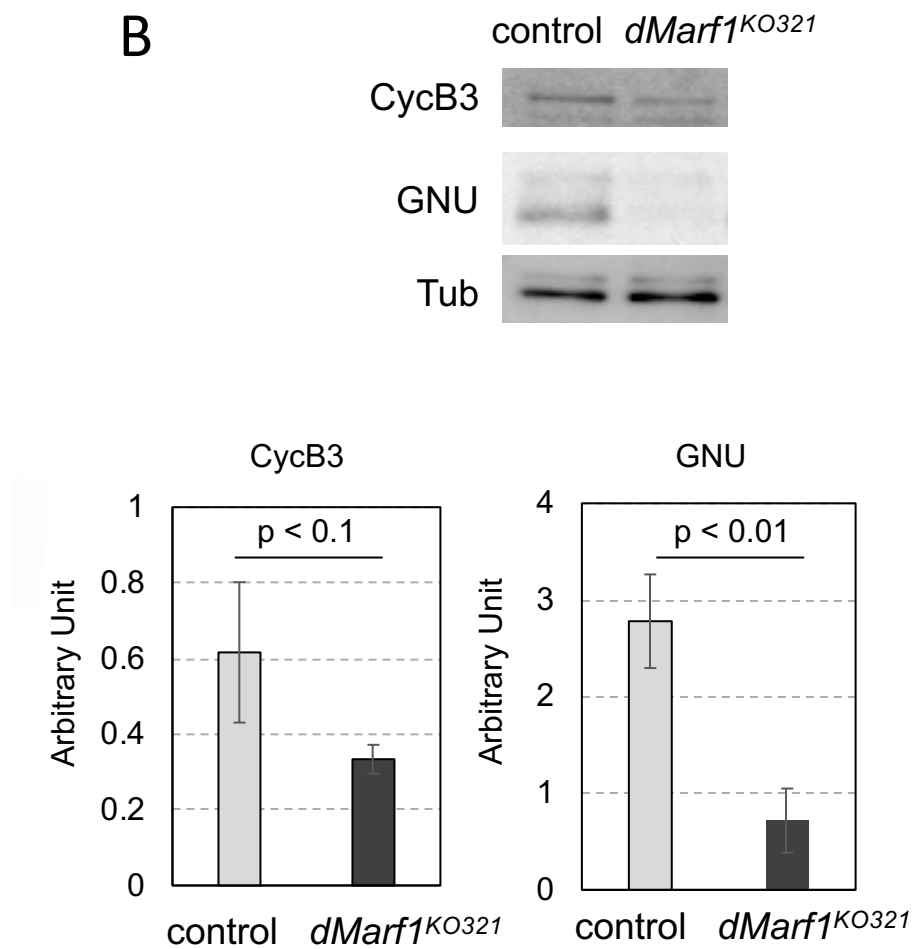

Supplement: S9 Fig — (A) Western blot showing protein expression at each stage of egg chambers in the heterozygous control and dMarf1KO321 females. (B) Quantitative analysis of CycB3 and Gnu protein expression in stage 14 egg chambers. The mean and standard deviation values are shown in terms of arbitrary unit. The p-value of the student’s t-test is also shown in the graph. (PDF) [file pone.0231114.s009.pdf]

Fig1B

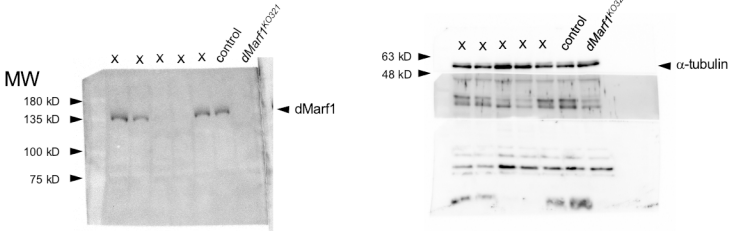

Fig1C

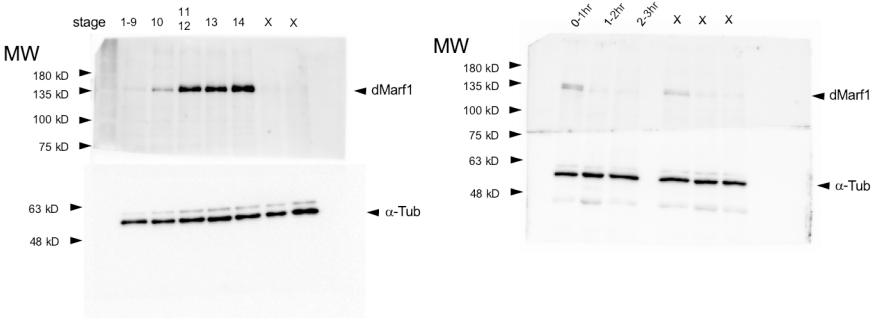

Fig5D

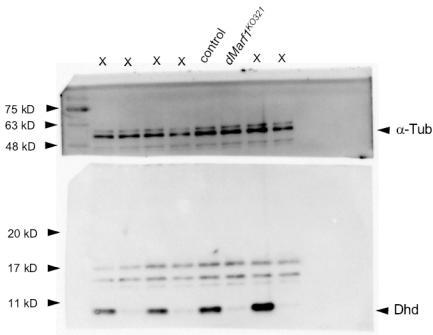

Supplementary Fig9A

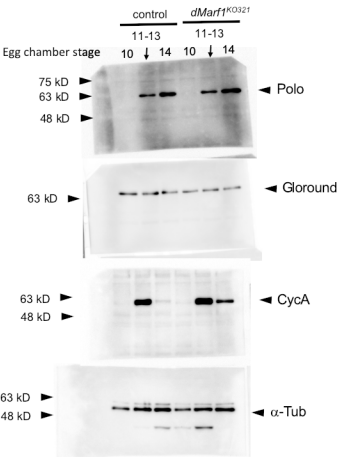

Fig6A

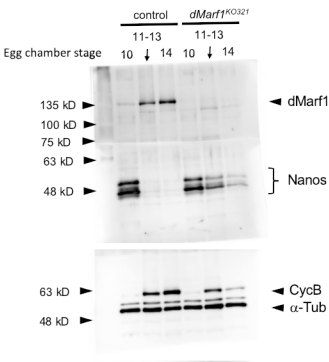

Supplementary Fig9B

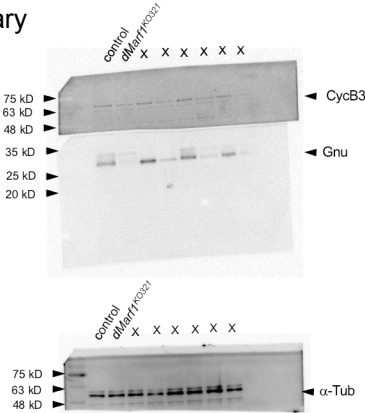

Supplement: S1 File — (PDF) [file pone.0231114.s016.pdf]
