# Supplementary material for: Drosophila MARF1 ensures proper oocyte maturation by regulating nanos expression
Source: PLoS One. 2020 Apr 3;15(4):e0231114. doi: 10.1371/journal.pone.0231114 (PMC7122799; doi:10.1371/journal.pone.0231114)

Supplementary figure 2

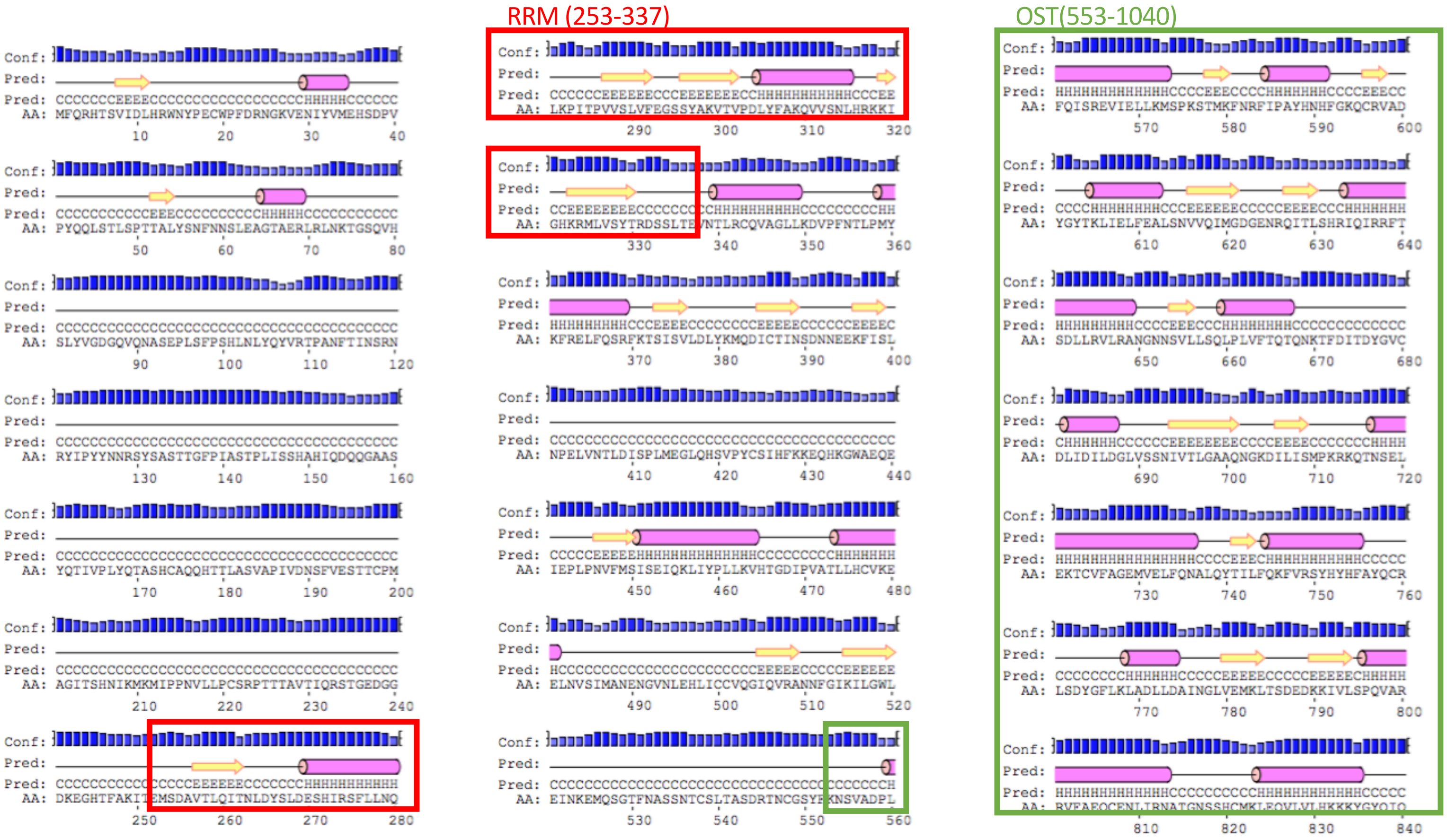

(continued from page 1)

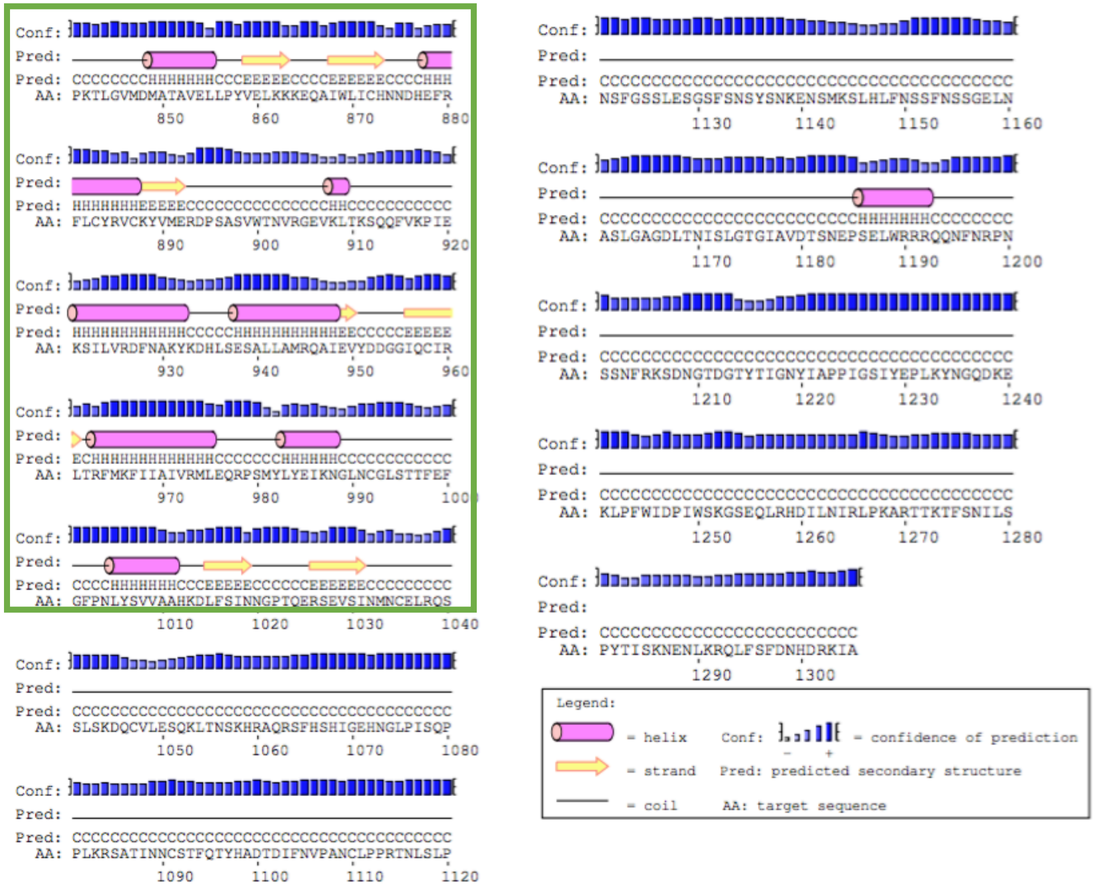

Supplement: S2 Fig — Secondary structure of dMarf1 polypeptide was predicted using the PSIPRED method. The vertical blue bar represents prediction confidence for each residue, and alpha helices and beta sheets are shown in pink barrels and yellow arrows, respectively. (PDF) [file pone.0231114.s002.pdf]
