# Supplementary material for: Drosophila MARF1 ensures proper oocyte maturation by regulating nanos expression
Source: PLoS One. 2020 Apr 3;15(4):e0231114. doi: 10.1371/journal.pone.0231114 (PMC7122799; doi:10.1371/journal.pone.0231114)
Supplement: S1 Protocol — (DOCX) [file pone.0231114.s015.docx]

**SUPPLEMENTARY INFORMATION**

**Generation of *dMarf1* mutant fly**

A loss-of-function allele of *dMarf1* was generated by targeting its coding region nearby the start codon with the CRISPR-Cas9 system (https://flycrispr.org/protocols/)[1]. The sense oligo, 5’-CTTCGGTCGCCGACATATAGGCTG, and the antisense oligo, 5’-AAACCAGCCTATATGTCGGCGACC, were annealed and ligated into the *Bbs*I sites of pU6-BbsI-chiRNA plasmids (Addgene, #45946). The resulted DNA plasmid was injected at 100 ng/µl into the dechorionated preblastoderm embryos containing Cas9 under *vasa* promoter (BL51324). The founder flies were crossed with *w^-^; Sco/CyO* flies to obtain F1 generation. The genomic region containing the potential mutation site of F1 flies was amplified and directly sequenced to determine the mutation or deletion sites.

1. Gratz SJ, Cummings AM, Nguyen JN, Hamm DC, Donohue LK, Harrison MM, et al. Genome engineering of Drosophila with the CRISPR RNA-guided Cas9 nuclease. Genetics. 2013;194: 1029–1035. doi:10.1534/genetics.113.152710
